# The “Schwarze Mander” of the Court Church in Innsbruck, Austria: Manufacture and Production of Monumental Brass Statues in the Renaissance

**DOI:** 10.1007/s40962-024-01299-4

**Published:** 2024-03-22

**Authors:** Marianne Mödlinger, Bastian Asmus, Giorgia Ghiara

**Affiliations:** 1 Institut für Archäologien, Universität Innsbruck, Innsbruck, Austria; 2 Labor für Archäometallurgie, Kenzinger Str. 32, 79336 Herbolzheim, Germany; 3 DISAT, Politecnico di Torino, Corso Duca degli Abruzzi 24, 10129 Turin, Italy

**Keywords:** brass casting, renaissance, statues, chemical analyses, X-ray Fluorescence, XRF, Principal Component Analysis, PCA, Maximilian I

## Abstract

The 28 "Schwarze Mander" (Black Men) are undoubtedly amongst the most magnificent monumental brass statues of the Renaissance. Commissioned by the Holy Roman Emperor Maximilian I for his funeral monument at the beginning of the 16th century, they were completed more than 30 years after the Emperor's death and are now part of his cenotaph in the Hofkirche in Innsbruck. Revising the original manuscripts and letters exchanged between Maximilian I and the various artists sheds light on the manufacturing process and the challenges the artists encountered whilst producing the statues. Moreover, the alloys used in the manufacture of the statues, now all blackened due to patination processes, were identified through non-invasive chemical analysis of all the statues.

**Supplementary Information:**

The online version contains supplementary material available at 10.1007/s40962-024-01299-4.

## Introduction

The 28 *Schwarze Mander* are without a doubt amongst the most stunning brass statues of the Renaissance period. They were commissioned by Maximilian I, the Holy Roman Emperor, for his funeral monument in the early 16th century but took over 30 years to complete after his death. Originally, the emperor wanted 40 statues of ancestors and saints of the Habsburg family and 100 statuettes of other saints associated with the House of Habsburg, as well as a sarcophagus for the emperor himself. In the end, 28 statues (Table 1), 23 statuettes and 34 busts of emperors (of which only 21 have survived) were created. The sarcophagus was replaced by a cenotaph with the kneeling statue of Maximilian I, the four virtues and 24 marble reliefs. Maximilian I, at the end, was buried in Wiener Neustadt.

**Table 1:** The 28 *Schwarze Mander* (Black Men) of the Hofkirche in Innsbruck, Austria

Statue no.	Name	Casting
1	Ferdinand II of Aragon (1452–1516)	1531
2	Joanna of Castile (1479–1555)	1528
3	Philip the Good (1396–1467)	1521
4	Charles the Bold (1433–1477)	1526
5	Cymburgis of Masovia (?–1429)	1516
6	Margaret of Austria, Duchess of Savoy (1480–1530)	1522
7	Maria Blanca Sforza (1472–1511)	1525
8	Sigismund, Archduke of Austria (1427–1496)	1523
9	King Arthur (mythologic)	1513
10	Ferdinand I (John) of Portugal (1345–1383)	1509
11	Ernest the Iron (1377–1427)	1512
12	Theodoric the Great	1513
13	Albert II of Germany (1397–1439)	1529
14	Rudolf I (1218–1291)	1516/1517
15	Philip I of Castile (1478–1506)	1514/1516
16	Clovis (466–511)	1550
17	Albert II, Duke of Austria (1298–1358)	1529
18	Frederick III (1415–1493)	1524
19	Leopold III, Margrave of Austria (?–1136)	1520
20	Albert IV, Count of Tyrol (1197–1239)	1518
21	Leopold III, Duke of Austria (1351–1386)	1519
22	Frederick IV, ‘Empty-Pockets’ (1382–1439)	1524
23	Albert I of Germany (1248–1308)	1527
24	Godfrey of Bouillon (1061–1100)	1533
25	Elizabeth of Luxemburg (1409–1442)	1530
26	Mary of Burgundy (1457–1482)	1517
27	Elisabeth of Carinthia (1263–1313)	1518
28	Kunigunde of Austria (1465–1520)	1516

In 1502, Maximilian I asked Konrad **Peutinger** of Augsburg, a humanist and scholar at the court of Maximilian I, to compile a list of all his direct ancestors, Roman emperors and saints, including their names and dates.^10^ This list was to form the basis of the monumental tomb of the emperor, who saw a statue of himself kneeling in front of his own tomb, surrounded and saddened by the statues of his ancestors. The statues, which were realised at the end, are listed in Table 2, together with an overview on the involved craftsmen. The creation of the Schwarze Mander in Innsbruck was not a single occurrence, nor were the castings completed by a single master or even a sole workshop. Their creation involved three generations and five different workshops. In this paper, only a broad overview can be provided.

**Table 2:** Overview on the Involved Artists (or workshops) in the Production of the 28 Statues (Chronological Order of Castings)

Statue	Draft	Casting (main)	Casting (later parts)	Sesselschreiber	Godl	Magt	Kölderer	Polhaimer	Dürer	Artus master	Erhart	P. Löffler	Vischer the Younger	Leinberger	Tiefenbrunn	weight (kg)
10	1508	1509	1514	1 2 3 5 6	5							4				1084
11	1513	1512	1518-20	1 2 3 4 6	5											1379
9	1513	1513							1	2			4			1005
12	1513	1513							1	2			4			1005
15	1514	1514/1516	1533	1 2 3 4 6	5 6											1267
5	1513	1516		4												1506
28	1514	1516		1 4 6										(2) (3)		1281
14	1511	1516/1517		1 2 3 4 6												1027
26	1511	1517	1528	1 3 4 6	5 6											1238
20	1513	1518			4 6				1					2 3		563
27	1514	1518		3 4 6 (son)					(1)	(1) (2)	(2)					1267
21	1513	1519			4	3	1									760
19	1519	1520			4 6	3	1									1224
3	1513	1521		1 2	4 6	3										1247
6	1513	1522			4 6	3									1 2	1281
8	1522	1523	1512	5	4 6	3	1 2									1267
18	1516	1524		(1)	4 6	3	(1) 2									1731
22	1513	1524		(1)	4 6	3	(1)								2	932
7	1512	1525		(1)	4 6	3									(1) 2	901
4	1513	1526		1	4 6	3	2									816
23	1513	1527		1	4 6	3		2								1370
2	1513	1528		(1)	4 6	3	(1) 2									1100
13	1526	1529			4	3		1 2								1368
17	1513	1529		1	4 6	3		2								1368
25	1513	1530		1	4 6	3		2								1416
1	1530	1531			4 6	2 3		1								1224
24	1513	1533		1	4 6	3		2								1267
16	1547	1550														1111

1 = Draft or *visierung*; 2 = Wooden model; 3 = Wax model; 4 = Casting; 5 = Casting (later parts); 6 = Post-casting treatment. The weight of the statues was calculated from the original Innsbrucker *Zentner*/hundredweight (1 cwt = 56.29 kg) and *Pfund*/pound (1 lbs = 0.5629 kg). Only the statue of Clovis (no. 16) was made after the draft and models of Arnberger and Amberger, and cast by G. Löffler; also, statue no. 5 (Cymburgis) was made after the design and model of Stoss and Rötlin (?) (After ^5,7,10,12^)

whilst there is an abundance of historical sources concerning the cenotaph in general, they predominantly focus on payments, contract details and other organisational matters. From merely a handful of passages can we glean any understanding of the technological aspects involved. We will refer whenever possible to the primary literature, i.e., the original sources from the 16th century (most of them can be consulted in the *Tiroler Landesarchiv* in Innsbruck, Austria) and their transcription (Regesten). The latter were published by the following authors: Regest 1-494^1^; Regest 496-2216^2^; Regest 2955-3031^3^; Regest 5693-5917^4^; Regest 6555-7938^5^ and Regest 9706-11495.^6^ Further Regesten were also published by Oettinger.^7^

In 1506, Maximilian I wrote to the *Räthe of the Raitkammer* in Innsbruck that they should build up the brass market and the *Rothschmied* (brass) foundry in Mühlau and invite the best brass makers from Nuremberg, the centre of copper-alloy casting in Europe. Tools and necessary metals were provided (Regest 811^8^). However, earlier the emperor already invited artists and craftsmen from Nuremberg and the surrounding area to Innsbruck. In 1502, the painter Gilg Sesselschreiber was hired by the emperor.^5^ Only a few years later, in Augsburg in 1508, he presented the emperor and Peutinger designs for the statues of the emperor’s grave. In the same year, Sesselschreiber moved to Mühlau/Innsbruck in order to start working on the statues. From 1510 on he had the sole responsibility for the production of the statues. His fully financed workshop included amongst others one painter, two (wax or wood) carvers, two casters (as well as his son Christoph, also a caster), one blacksmith and one brass fettler (*Ausbereiter* or *Ausberaiter*), a smith as well as sometimes a gold smith (Regest 970; 1055; 1101). Soon afterwards but latest in 1514, his stepson Wolfgang Teininger and his son-in-law Sebastian Häuserer followed as wax carvers. However, as not all the necessary knowledge seems to have been available, the casting of the first statue, John I of Portugal (no. 10), took place only with the help of the bell caster and gun founder Peter Löffler in 1509 (Regest 949). Peter Löffler (also Laminger or Laiminger) is the progenitor of the famous family that dominated metal casting in Tyrol until the 17th century. It is worth noting that the statue of John I of Portugal (1357-1433) has been associated with his half-brother, Ferdinand I of Portugal, since 1513 for reasons still unknown;^8^ until today, the statue is generally associated with Ferdinand I (as in the following in order to avoid confusion).

Most likely hired directly by the emperor in 1512, Albrecht Dürer is responsible for the design of the following statues: King Arthur (no. 9), Theodoric the Great (no. 12), Albert IV, Count of Habsburg (no. 20) and the not anymore preserved statues of Charlemagne and Ottobert, son of Theodobert.^9,10^ In 1513, when Gilg Sesselschreiber did not complete his work as planned, Maximilian I asked the brass founder Peter Vischer of Nuremberg to cast the statues of Arthur (no. 9) and Theodoric the Great (no. 12), which were cast in the same year (Regest 5793). These two statues were the very same year pledged to the bishop of Augsburg and could be bought back by Ferdinand I only in 1531.^5^ The emperor also asked Veit Stoß in Nuremberg in 1514 for designing and modelling further statues. Unfortunately, Stoß did not succeed due to a boycott of local casters. Also Hans Leinberger, who was asked to cast one of the statues (Regest 1166 und 4023), resigned after he did not manage to cast even small objects successfully. His model of Albert IV, Count of Habsburg (no. 20), which was created after a design of Albrecht Dürer, was cast only years later, in 1518 (Regest 1322), by Stefan Godl from Nuremberg, who was already appointed by the emperor for other works in 1508 (Regest 909). From 1518 onwards, Jörg Kölderer (painter and master builder) was responsible for designing the statues.^5^

With only two statues cast in 1515, and Sesselschreiber's constant requests for further funding, supposedly for the production of the statues, Sesselschreiber fled Innsbruck, was imprisoned in Augsburg and only released when his son and son-in-law promised to complete all the statues ordered by 1516 (Regest 1245). However, only eight out of eleven cast statues were (more or less) cast. In 1518, the emperor dismissed Sesselschreiber and appointed Godl as head of the workshop and responsible for the casting of statues and statuettes. The foundryman from Nuremberg was called to Innsbruck already in 1508 in order to establish the "*handwerch der rodsmiederey*" (craftmanship of brass caster and worker) (Regest 923). His casting of the statue of Albert IV, Count of Habsburg (no. 20), at his own expense, from a model by Hans Leinberger, earned him the commission for the remaining statues in June 1518 (Regest 1322).

**Figure 1:**
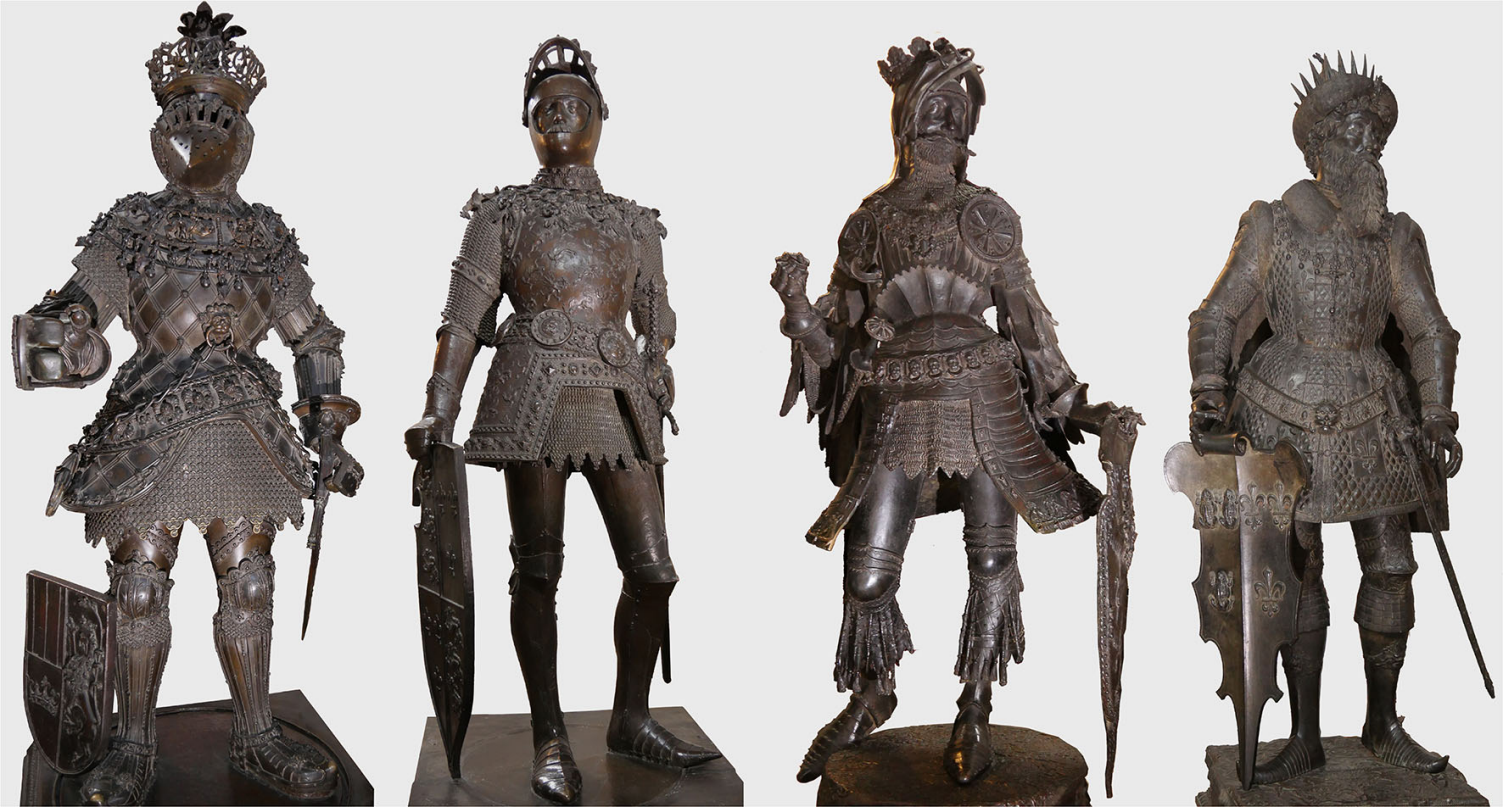
Selected statues from the most important workshops (from left to right): Ferdinand I. Portugal (no. 10; cast by Sesselschreiber and Laiminger); King Arthur (no. 9; cast by Vischer); Albert IV (no. 20, cast by Godl); and Clovis (no. 16; cast by G. Löffler) (© Innsbruck, Tiroler Landesmuseen, Hofkirche. Foto & editing: M. Mödlinger)

The sudden death of Maximilian I on 12 January 1519 put a stop to the funding and thus to the work of the workshops engaged in the production of further statues. Only with the help of Jörg Firmian, marshal of the regiment in Innsbruck, did the work continue in 1521 (Regest 1376). The statue of Philip the Good (no. 3) was completed in the same year. Ferdinand II, Archduke of Austria, decided in 1523 that two statues should be cast each year (Regest 1479). In 1527, he asked the Innsbruck government to hire Jörg Kölderer for the visualisation of the whole funeral monument (Regest 1730); Kölderer thus created a model of the monument, using the original measurements of the statues, provided by Godl. In 1522 he drew up drawings of the existing and still missing statues.^11^ By 1528, eleven more statues had been cast (Figure 1).

After Godl took over the main responsibility for casting the statues in 1518, he made the models for 16 statues based on designs by Kölderer and Polhaimer. Unfortunately, Melchior, Godl's brother and colleague, died in 1530, and Godl suffered from severe health problems; as a result, almost no work was done until 1531. A year later Leonhard **Magt**, the wood and wax carver, died (Regest 1916). Not much is known about Magt; he was not from Tyrol and learned to work with wax whilst working for and with Godl. In 1532 and 1533 only one statue a year was cast under the responsibility of Godl's cousin, Bernhard Godl. Stefan Godl died as a result of his health problems in March 1534 (Regest 1956). He was responsible for the casting of 17 statues and 23 statuettes.

In 1545, at the Imperial Diet of Augsburg, Christoph **Amberger** (painter, drawer, graphic artist) was commissioned by Ferdinand II with the designs for the missing statues, of which eight designs have survived and only the first was realised: Clovis (no. 16), Ottobertus, Charlemagne, Gisela of Hungary, Stephen I of Hungary, Radbot of Klettgau, Viridis Visconti, and Hugh the Great (for reproductions see^10^). More or less the same time, Veit **Arnberger** (sculptor, medallist, stamp cutter) was employed at Löffler's foundry in Innsbruck. He is known to have modelled the statue of Clovis, which was designed by Christoph Amberger of Augsburg and cast by Gregor **Löffler**, (caster, gun founder), a son of Peter Löffler, who was one of the most renowned artillery experts - in terms of production - in 16th century Europe. Work on the statues was only resumed in 1548 by order of Ferdinand II. In 1550, the only statue of Gregor Löffler, and the final one for the funeral monument, was finished (Clovis; no. 16). Löffler refused to cast any more statues because of his poor health. In 1565, in his last will and testament, he warned his sons not to continue casting statues (Regest 10103).

Several more statues seemed to have been cast but melted down at a later point:

1. Amberger made the draft/*visierung* for the statue of Charlemagne before 1551, which was cast by Gregor Löffler in 1551 (Regest 6928), but melted down again in 1569.^8^
2. Eleonora, made of copper by Sesselschreiber, was included in the inventory by Kölderer in 1528 (Regest 3011), and again by Godl (Regest 3010); Kölderer notes:
  *“ist auch von kupher auf das allerschlechtest geformbt und auch vast ubel gefallen, hat kain guidein stuckh in iren claidungen, kain cron, kain halspant, kain clainet, ain pewrische prust, kain zier daran, ist in sehen löcherig”*
  She is also made of copper and is very badly moulded and ugly, has no golden piece on her clothing, no crown, no necklace, no jewel, a peasant breast, no ornament on it, and is full of holes.
3. Ladislaus was cast in copper by Sesselschreiber and listed in the inventories of Kölderer and Godl in 1528 (Regest 3010 and 3011);
4. Theodopertus, cast by Sesselschreiber, was described by Godl in 1528 (Regest 3010) and melted latest in the 17th century:
  *ist dises pild am leib vast löcherig, das haubt auch ledig, der part nit wol gefallen*
  This depiction is almost full of holes in the body, the head is also bare, that part [beard?] does not please
5. It seems as if there was also a statue of Dietrich from Bern (Theodoric), which was not appreciated by the emperor Karl V; consequently, as noted by Georg Füger in 1548, one might cast another statue from this one, or provide it with another name (Regest 6398).

Hans Christian Löffler, the son of Gregor Löffler, complains in 1568 in a letter to the government that he still has besides the standing statue of Chlovis two other statues laying around in his workshop and hindering his work: they should be kindly taken away. It is not clear which statues those “*zwai gegossene pilder”* [two cast images] are (Regest 10154).

## The Workshops and the Production of the Statues

**Figure 2:**
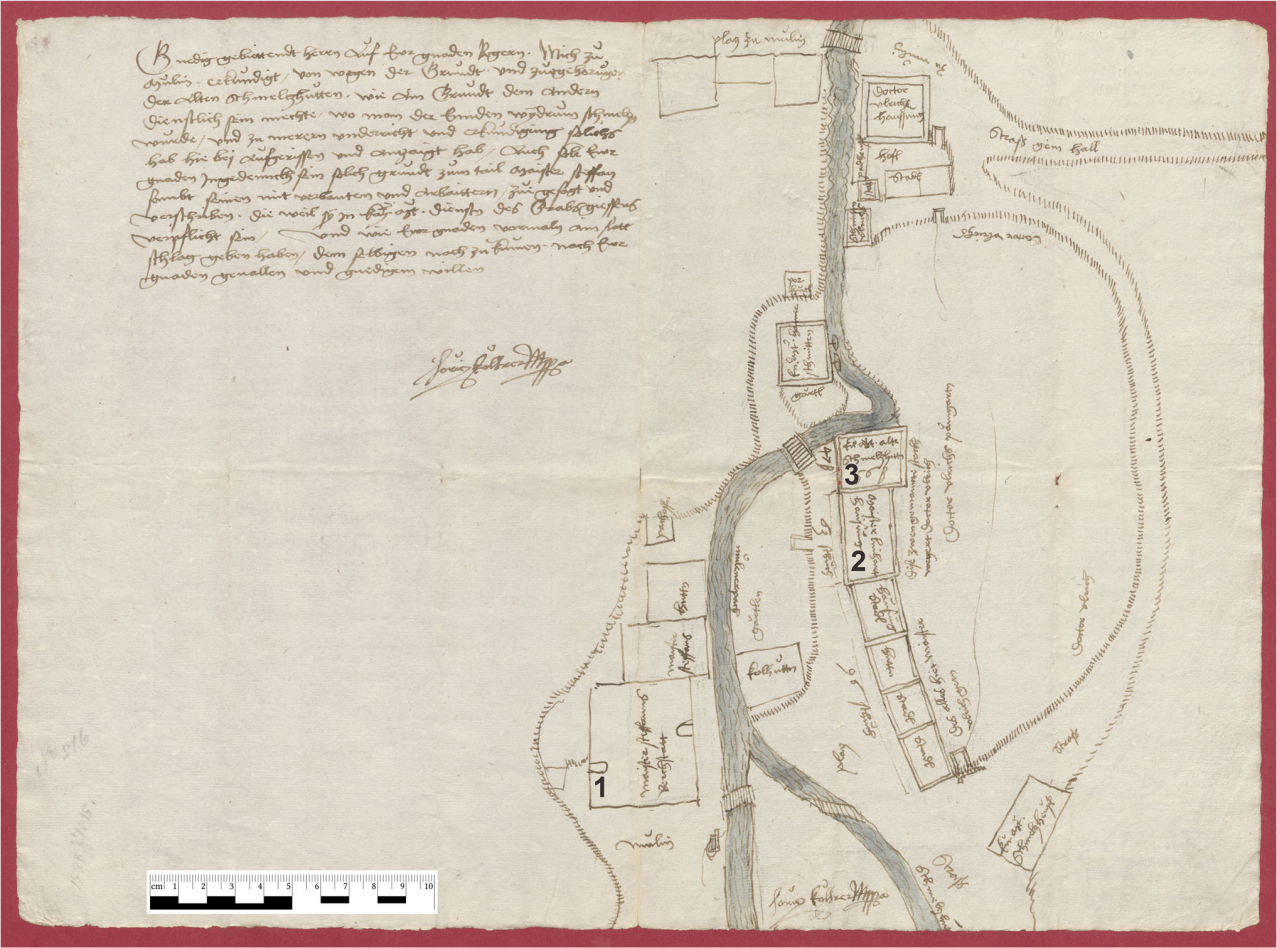
Map of Mühlau from Kölderer (1508), modified around 1530 (after: Tiroler Landesarchiv, Maximiliana XI. 68). The numbers indicate the workshops involved in the production of the statues. 1 - Godl; 2 - Magt; 3 - Sesselschreiber. Today, only building no. 1 has been preserved (Ferdinand-Weyrer-Str. 3); another building, built in 1511, to the south of building no. 1, has also been preserved (Ferdinand-Weyrer-Str. 1). It was built as a storehouse and living quarters for the craftsmen.^13^ Source: Tiroler Landesarchiv, Maximiliana XI. 68.

Metalworking was booming in 15th century Tyrol due to the rich ore sources and excellent craftsmen: iron processing in the Stubai-valley, armoury and plating workshops in and around Innsbruck, the copper industry in the Ahrn valley and Brixlegg, the silver industry in Schwaz and the brass workshops in Kramsach and Mühlau were amongst the most important ones. In Mühlau close to Innsbruck three imperial foundries and smelting works existed. *Schmelzhütte*, refers to smelting works or casting workshops, or more generally metallurgical works, incorporating also silver extraction by cupellation: one for silver, one for copper/brass and one for gunsmiths and armourers. Stamp or pounding mills for the production of clay/loam were also present. It was the aim of Maximilian I to establish the *rothsmiederey,* a school of brass working in Mühlau, so he tried to bring the most renowned brass caster and workers from Nuremberg to Tyrol (see above). A map designed by Kölderer in 1508 andmodified around 1530 depicts Mühlau with its various metal and metalworking related workshops (Figure 2), amongst them also the workshops of Sesselschreiber and Godl who at that time worked independently next to one another, as well as Magt.

In the early 16th century, there was a limited tradition of casting large sculptures north of the Alps. The desire to do so was inspired by the ideals of the Renaissance. Founders and sculptors of the era had limited familiarity with the intricacies of casting life-size or larger sculptures, yet they possessed significant expertise in handling substantial quantities of liquid metal in vocations such as bell-casting and cannon-making. We must explore how these associated trades impacted the sculptures produced by the Mühlau casting workshop.

Our knowledge of **workshop equipment** is limited to the inventories commissioned by the emperor in 1513 (Regest 1101 and 1137) and a few more notes. The first inventory only lists "*werchzeug, wachs, kupfer, messing, holz, kol, gepew*" [tools, wax, copper, brass, wood, charcoal, component/building] without further explanation, whilst the latter, detailed in Table 3, provides more extensive information. In 1509, the emperor states that Sesselschreiber will need for his workshop copper, brass, iron, wax, coal, wood and other things (Regest 975). Additional information found in other publications, such as "two crucibles per furnace,"^12^ is fabricated.

**Table 3:** The Inventory of the Casting Workshop from 1513 (Regest 1137): on the Left in Early New High German; on the Right the English Translation

Regest 1137 (1513)	Regest 1137 (1513)
*Allerley zeug.*	*All sorts of things.*
Item sechs eisine getter, darein man die schilt formbt.	Six iron grilles, in which shields are moulded [loam moulds were reinforced with iron bands and bars. It seems very likely they had some grilles prepared for recurring tasks such as casting the shields]
Item drewhundert Nuremberger tögl.	300 Crucibles from Nuremberg
Item ain zugerichten hamer an das wasser gericht.	one water powered hammer
Item ain stampf dabey, darein man rotten laym vnd anders innen stempft.	one tamping mill, in which rotten loam and other materials are tamped [fermented loam]
*In der schmidten.*	*In the smithie*
Item ain zugerichte ess mit zwai pelgen.	One smithing hearth with two bellows
Item ain grossen vnd ain klainen hornampass.	One large and one small horn anvil
Item acht eisine schmitzangen.	Eight iron forging tongs
Item zwelif stuck maistl, fürschleg vnd werchhämer.	Twelve chisels, sledgehammers and hammers
Item sechs fueder kol.	Six carts of charcoal [volume measure, in Tyrol some 600 to 800 l] one wheelbarrow
Item ain laufkarren.	
Item anderhalben puschen eisen.	One and a half bundle iron [lit. tuft, bundle seems more appropriate]
Item ain grosse eisene wag, on gewicht.	One large iron scale, without weights
Item allen werchzeug zum ausberaiten.	All tools for fettling and chasing
	*On the statues (selected)*
Item fraw Margret, kais. mjt. tochter, ist von holz geschnitten vnd mit rupfein leinbat überzogen.	Item lady Margret, daughter of His Imperial Majesty, is carved from wood and covered with [rough, raw] linen cloth
Item herzog Ernst von Osterreich ist geschnitten von holz vnd possiert.	Item Duke Ernest of Austria, is carved from wood and [posed, modelled, sculpted]
Item ain hülzeins geschnitten prustpild, fraw Zira von der Mass, kais. mjt. Muter	Item a wooden carved bust portrait, Lady Zira of Masovia, His Imperial Majesty's mother [sic!]

Comments by the authors in […].

It remains to be said that it is highly desirable to carry out a documentation of the history of the building, a geophysical survey and possibly an excavation of the ground floor of the building in order to obtain further important information about the foundry in the building, its organisation and equipment.

### The Source of Raw Materials

In the following, we present a short overview on the few documents which provide further details about origin, quantity and price of the raw materials, such ascopper, wax, loam and crucibles used for the production of the statues. As far as reconstructable from remaining documents, the **copper** used for the casting of the statues derived exclusively from local Tyrolian mines such as Prettau/Southern Tyrol, the famous “Tauferer copper” (Table 4). However, we do not have any documentation about the origin of the Calamine, thezinc ore for the production of brass. Brass was created through cementation as zinc in its metallic form was not yet discovered.^14^

**Table 4:** Copper and Brass Ordered and Used for the Casting of the *Schwarze Mander*

Regest	Year	Caster	Material	Quantity (cwt / lb)	Cost	Cost (h)	Comments
1101	1513	SE	Brass	111 / 18	598 fl, 49kr	5fl, 23kr	The list of expenses by the Sesselschreiber workshop notes the expenses for brass made of Tauferer copper
1225	1516	SE	Tauferer copper	?			The emperor sold to the Hochstettner from Augsburg the “Tauferer mine” for 5 years with the obligation to provide him with about 11.2 t (200 cwt) copper per year for his armoury (Zeughaus) and funeral monument
1247	1516	SE	Copper	?			Maximilian I notes that Jacob Fugger promised to lend copper to Gilg Sesselschreiber, for which he should be paid
1263	1517	SE	Copper	30 / 0			Maximilian I ordered from the foundry of Fugger in Schwaz copper for Sesselschreiber’s workshop
1285	1517	SE	Copper	150 / 0	825 fl	5fl, 50Kr	Payment for copper to the Fuggers in Schwaz
1339, 1340	1518	GO	Brass	18 / 75	112fl, 30Kr	6fl	Godl received brass (*stück möss*) from Landshut, for casting Albert IV, Duke of Austria (no. 17) and likely another statue
1373	1520	GO	Copper	25 / 0			Godl received copper
1450	1522	GO	Copper	40 / 0			The *Hutmeister* in Rattenberg is urged to provide copper for the casting of the statues in Mühlau
1576	1525	GO	Tauferer copper	25 / 0			Godl received Tauferer copper
1717	1527	GO	Copper	25 / 0			Godl received Tauferer copper for casting King Albert II of Germany (no. 13)
1761	1528	GO	Copper	50 / 0	212fl, 30kr	4fl, 15Kr	The chamberlain Narciss Stoppl is commissioned to pay Wolf Vittl instead of the Hochstetters of Augsburg 50 hundredweight of copper, which the latter had handed over to Godl in 1527 and 1528
1771	1529	GO	Tauferer copper	25 / 0			Godl is supposed to receive Tauferer copper
1829	1530	GO	Tauferer copper	25 / 0			Godl is supposed to receive Tauferer copper for casting Elizabeth of Luxemburg (no. 25)
1915	1532	GO	Tauferer copper	25 / 0			Godl is supposed to receive Tauferer copper from the armoury
1921	1533	GO	Tauferer copper	"Fass Kupfer"			A “Fass Kupfer”, a barrel of copper, has to be brought directly from Taufers to Mühlau
6783, 6787, 6850	1549	LÖ	Brass (scrap)	34 / 0			Gregor Löffler received from the Innsbruck armour old hackbutts and handgonnes made of brass for casting Clovis (no. 16) and Charlemagne

*Cwt* *Zentner*/hundredweight, *lbs* *Pfund*(pound); *fl* Gulden, *kr* Kreuzer, *f* Vierer; *GO* Godl, *SE* Sesselschreiber, *LÖ* Löffler

The prices for copper and brass fluctuate somewhat (Table 4), but generally are 4 fl and 40 kr per cwt and another 1 fl, 30 kr for turning it into brass. According to^15^ the Gulden (fl) has 60 Kreuzer (kr), whilst five Vierer (f) make one Kreuzer.

The sources (Table 4) tell us about the metal supply for the Sesselschreiber and Godl workshops. However, there are some gaps in the data for Sesselschreiber. Over the years, Sesselschreiber received the substantial amount of 16.3 tonnes of copper and brass, but the surviving cast objects only amounted to approximately 7.8 t (Table 2). Three statues were melted down again over the years, so we may add another 4 t of metal to the bill of materials he used for the emperor's grave, adding up to some 12 t of metal. The inventory of 1516 lists 13 cwt, where there should have been 50 cwt, according to the *Raitkammer’s* records. But even those were claimed by Sesselschreiber’s son and son-in-law to be a private stock (Regest 1250). Considering a very generous metal loss of 10% (Regest 10307), the amount of metal that Sesselschreiber used for casting was around 13.2 t. This leaves a discrepancy of about 3 tonnes, which cannot be explained technically. It is possible that the records are incomplete, but it is equally possible that the metal was used for other purposes than intended by the emperor.

The picture is slightly clearer for the workshops of Godl and Löffler. As the statues had to be weighted in order to calculate the caster’s salary, we also know their weight and can relate it to the quantity of copper received. In two cases, we can relate their weight directly to the amount of copper received for the casting of the statues:

The statue of Albert II (no. 13) weighs about 24.3 cwt including the base and that of Elizabeth of Hungary (no. 25) about 25.15 cwt including the base. The actual casting weight of course was higher than that, as we have to take account of the gating system or the sprue which had to be chiselled off (we assume here about 5% of the final weight of the statue). We know that Godl received for each statue 25 cwt. copper. Obviously, zinc was added in the form of calamine during the cementation process that turned copper into brass. We can assume a resulting brass composition with 25–30 wt% zinc^16^ to which copper hat to be added to achieve the final chemical composition of each statue. The statues contain 15.4 wt% (Albert II) and 15.5% (Elizabeth) zinc. Assuming that Godl used all of the copper provided for these two statues, we can calculate the average metal loss during the melting process: For Albert II, this would be about 11.5% and for Elizabeth 8.5%.

**Wax** is often mentioned in the documents, but usually as part of complaints, or that it is missing and urgently needed (for example: Regest 949; 975; 1100; 1336; 1344; 1449; 1883). However, on some occasions it is mentioned when describing the progress in the casting of certain statues (Regest 1137, 1772). In 1513 the government agency dealing with finances, the *Raitkammer* presented Maximilian I with the bookkeeping records regarding the costs for equipment, consumables and salaries for Gilg Sesselschreiber and his workshop (Regest 1101). It contains details on the acquisition and the price of wax between 1510 and 1513 for about 122 fl and 29 kr: In 1513 not only the price but also the mass of wax obtained is reported: 2.5 cwt for 39 fl, 5 kr putting the hundredweight of wax at 15 fl 40 kr. Assuming the cost of wax remained the same throughout these three years, Gilg Sesselschreiber bought about 7 cwt and 73 lbs of wax, which equals some 435 kg.

The wax amount, either volumetric or according to its weight can be used to determine the amount needed for casting, however we cannot prove the workshops did this. As a rule of thumb, the mass of an object cast in a copper alloy is 10 times the weight in wax, which is a simple ratio of their respective densities. In other words, 435 kg of wax equals some 4.3 t of cast object in a copper alloy. Above we laid out that Sesselschreiber cast statues weighing approximately 12 t altogether. This suggests that wax was recycled in the workshop, which is neither unheard of nor surprising, but fascinating that we have written records detailed enough to tell us as much.

The little information we have about the **clay or loam** used to create the moulds for the casting of the statues is from a complaint of the carver Magt about his employer Godl in 1529 (Regest 1772).The loam is not prepared as it should be, it is still green from the mountain, so the mould is coarse and impure (see also below). From the 1513 inventory we know that the loam was fermented in order to improve its plasticity (Regest 1137):

*“item ain stampf dabey, darein man rotten laym vnd anders innen stempft* "

This translates to: there is a tamping mill in which rotten loam and other materials are tamped. Apparently the loam was left to rot, that is to say to be fermented for a couple of weeks before usage. This is also common for bell foundries, such as the Tyrolean Grassmayr foundry: beer, yeast and molasses were used to ferment the loam. amongst the last foundrymen to cast statues is Lendenstreich in 1571. He offers a bill of materials for the preparation of the moulding loam; it contains iron wire, wool clippings and calves hair (Regest 10307).

In 1513 (Regest 1137), the foundry inventory listed also 300 **crucibles** from Nuremberg. Also Godl ordered crucibles from Nurnberg, which arrived in 1518 (Regest 1344). Another order for *häven vnd tegel* [pots and crucibles] for casting was placed in Nuremberg by Godl in 1525 (Regest 1575). In 1527, one more order was placed in Nuremberg, this time directly from *Regiment und Kammer* in Innsbruck for a number of *häven vnd tegel*, which master Stefan Godl, caster in Mühlau, needs to buy “*zu notturft eins grossen geschnittnen pilds”* (urgently for a big, cut image; i.e. for the casting of a statue). Also in 1529, another order for crucibles was placed in Nuremberg (Regest 1802). Unfortunately, the sources do not clarify the size of these crucibles. Despite claims that the Nuremberg crucibles were made of graphite,^10,12^ these assertions are baseless. The sources clearly indicate that the crucibles were actually made of clay from the nearby Heroldsberg clay pit (Regest 5743), with no mention of any graphite deposits in or around Nuremberg. The availability of this raw material was an important factor in Nuremberg's location, and the brass industry heavily depended on it. Highly refractory materials were a scarce commodity and not readily shared, even with the emperor Maximilian I, who encountered significant challenges in procuring the required raw materials (Regest 5740-5753 for the difficult process of obtaining the desired clay from Nuremberg).

### The Furnaces of the Workshop

According to the inventory from 1513 (Regest 1137), the foundry of Sesselschreiber had 16 crucible furnaces with *den grossen giesofen* (the big casting furnace) situated in the centre of the hut, as indicated in the inventories. There is just one piercing iron (see below) noted, as only one large *giesofen*. The picture of Sesselschreiber's son Christof shows the large palgofen with a taphole. Knitel^12^ suggests that the *giesofen* was a kiln used to remove wax from the clay/loam moulds and bake them. However, there is no supporting evidence for this claim. Agricola’s work^17^ (Chapter XI, *Vom Entsilbern des Kupfers*) is utilised to support Knitel's hypothesis. The presence of the picture of the liquation furnace in Knitel's work prompts queries about its pertinence to his hypothesis. Although it is feasible that such a furnace could be utilised for processes related to moulds, Knitel's hypothesis lacks explicit discussion or justification for this selection. Moreover, the assumption that casting procedures in the 16th century emulated contemporary foundry practises could disregard the subtleties of historical craft traditions and technological advancements. Furthermore, failure to disclose the motive behind the image's use could result in misinterpretations by the reader.

**Figure 3:**
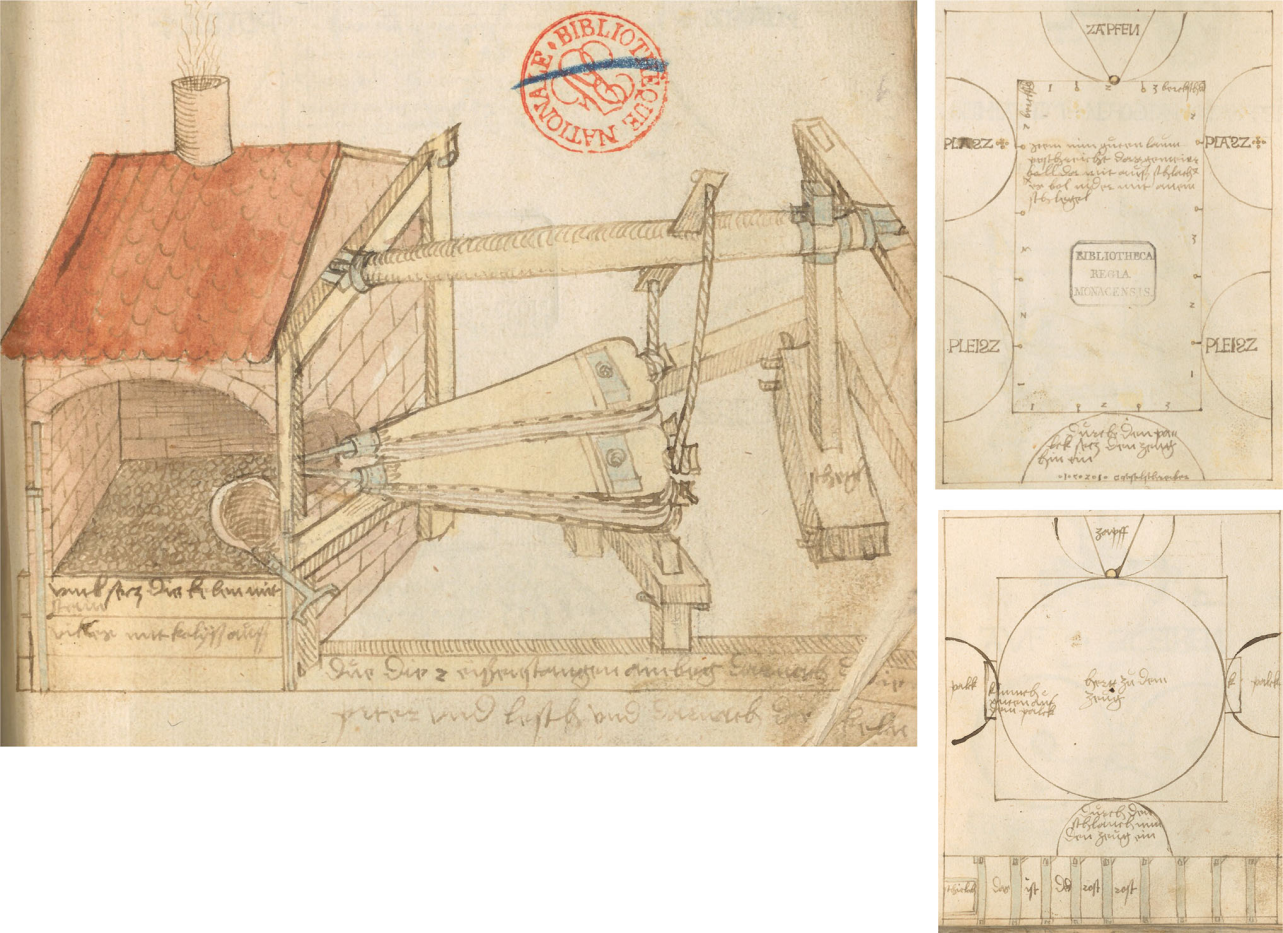
From the manual of C. Seselschreiber 1524^18^ (CC BY-NC-SA 4.0 Deed). Left: *Palgofen mit ainer kelen*. Top right: the *Palg Ofen* (a large furnace operated by six bellows). Below right: the *Wintofen*, a reverberatory furnace.

It is much more probable that the large casting furnace, mentioned in Regest 1137, is a furnace designed for melting large amounts of metal. Gilg Sesselschreiber’s son Christof shows three contemporary furnace designs in his book on cannon and bell foundry;^18^ A bellow driven furnace, where metal is melted in a ladle, is called “*Palgofen mit ainer kelen*”, a large furnace operated by six bellows, is called a “*Palg Ofen*'' and a reverberatory furnace, is called “*Wintofen*” by Christof Seselschreiber (Figure 3).

There is a highly instructive item in the inventory from 1513 (Regest 1137) (Table 3), which furthers our understanding of the kind of furnaces that were present in the workshop:

“*Item ain ansticheisen.*“

“Item one piercing iron”

This is a **pricking or piercing tool** for tapping a furnace. Crucible furnaces do not require tapping; a piercing iron is only needed in a furnace that needs tapping, so the large casting furnace in the inventory is either a reverberatory furnace or the *Palgofen*. Seselschreiber shows a bellow driven, charcoal fired furnace, where the metal is in direct contact with the charcoal. A similar furnace design is already mentioned in Theophilus Presbyter’s *schedula* in the bell casting chapter,^19^ and is likely the design used in Sesselschreiber’s workshop, as there is ample mentioning of charcoal, but not so much of firewood, which is needed for a reverberatory furnace.

It might be worth to expand here on the terminology used and briefly describe the different types of ovens used at the beginning of the 16^th^ century:

1. The **crucible furnaces** are only mentioned as furnaces in the inventory of Sesselschreiber’s workshop. There are sixteen furnaces. We do not know exactly what they looked like, but the following considerations have been made: 300 crucibles from Nuremberg are listed, as well as "six tongs for lifting the crucibles from the furnaces". This tells us not only that these were crucible tongs, but that they were used to lift the crucibles out of the furnaces. We can also assume that a maximum of six tongs were in use at the same time: the crucibles were lifted more or less simultaneously or in short succession. We can prove that three crucibles can be pulled in quick succession with one crucible tong and still produce a successful casting. There is therefore no reason why it should not be possible to run 16 furnaces simultaneously for a large casting, although this would require a very experienced and dedicated team. It is quite possible that these furnaces required forced air through bellows, but it is also possible that these furnaces used only natural draught. Both types of furnace are known to have coexisted at least from the time of Theophilus.^20^
2. The **Palgofen** is literally a bellows stove, i.e. a stove driven by bellows. A drawing by Sesselschreiber's son Christof shows six bellows and a taphole. In its main technological features it seems to be a continuation of the medieval smelting technology as described by Theophilus Presbyter in his chapter on the foundation of bells, where he describes a comparatively large structure capable of melting about 400 kg,^20^ supplied with air by a pair of large bellows, operated by two men. The melting charge is mixed with the fuel, which is 'charcoal', and has to be tapped for casting.
3. The **reverberatory furnace** is a radically different concept to the two above. It used wood as fuel and melted the metal with the tips of the flames passing over the metal. It was the predecessor of the puddle furnace and appeared more or less suddenly in Europe at the end of the Middle Ages.

A significant change occurred in production some 20 years after Sesselschreiber's and Godl's work. In 1550 (Regest 6850), Gregor Löffler reported a loss of over 4 cwt of metal in the fire, resulting in the calculation of 25 cwt of metal used. As zinc sublimates at 907°C and quickly oxidises to form zinc oxide, significant amounts of zinc are lost rapidly. Additionally, the production and casting of brass is hazardous due to the toxicity of zinc oxides, as noted also by Gregor Löffler’s son Hans Christof (Regest 10103):

“*Dann die arbait mit dem wachs und messing im feur ain solches ungesunds werk ist, das niemand glaubt, als wer dasselbig im werk erfert.*”

“working with wax and brass in the fire is such an unhealthy work, [but] no one believes it, who has not worked it himself.”

However, crucibles were no longer used, and the metal was melted directly in the furnace, as noted by Gregor Löffler (Regest 6805):

“...*Den messing in den Öfen zu schmelzen ist treffenlich streng, dermassen dass wenig maister befunden werden, die den messing mit Öfen frei wie ander metall schmelzen kunten sondern müssen den nur in teglen schmelzen und meistern.*”

..to melt the brass in the [reverberatory] furnaces is truly difficult, to such a extent that few masters are to be found, who are able to melt the metals freely like other metals, but have to melt and master it in crucibles.”

The loss of almost 16% is a significantly greater metal loss compared to other recordings, typically estimated at around 10%, as noted by Hans Lendenstraich (Regest 10228 from 1570 and Regest 10307 from 1571), and may be explained with the adoption of a new technology.

Gregor Löffler, the most prolific gun founder of the period, may well have used a reverberatory furnace here. As a gun founder, he needed to melt large quantities of metal on a regular basis. A task that would have been much easier with the modern reverberatory furnace compared to the mediaeval technique of melting the metal with bellows in charcoal. Charcoal requires a lot of labour and is more expensive to use.

A reverberatory furnace (Figure 3**, right**) uses firewood, especially soft woods with a high proportion of essential oils, such as spruce and fir. The metal is melted by the flames alone, which are forced onto the metal in a domed furnace. Graphic representations of this type of furnace appear in Italy in the early 16th century, most notably in Leonardo da Vinci's Codex Arundel. From the mid-16th century they appear in the Italian metallurgical treatises of Biringuccio^21^ and Cellini,^22^ both of which include detailed descriptions of various bronze casting techniques, and none of which seem to have been applied in the case of the casting of the black men.

### The Production of the Statues

#### Introduction

The cenotaph has been extensively researched since the late 19th century, resulting in a vast body of literature on its history (such as^5^), iconography (for instance^10,23^), design, and the individuals and workshops involved in its creation.^7^ Efforts were made to differentiate between creative artists, sculptors, and foundry workers, leading to a lengthy art-historical discussion regarding the identification of individual masters and artists. Although this is a conventional art-historical approach, examining the manufacturing processes in detail was not received enthusiastically. The only significant research on related casting procedures was conducted by Oberhammer.^10^ It is necessary to reassess the manufacturing process considering the progress made in archaeometallurgy and the history of technology over the past 80 years. As mentioned earlier, our focus will be on the primary sources - specifically, the original sources from the 16th century and their transcriptions (Regesten) - whenever possible. This choice is driven by the potential for misinterpretation or the introduction of conjectural ‘evidence' by authors of secondary literature from the 19th or 20th century.

The sources indicated in Table 5 are the only ones providing us with direct information for reconstructing the production process of the statues. The texts can be divided into three groups: those that are contemporary with the production of the statues and report on the processes themselves, such as Regest 949 and 1772; those that require deductions based on listed objects or materials, of which the inventories of Sesselschreiber’s workshop from 1513 and 1516 are the most significant (Regest 1137 and 1250), and finally those texts that report on some aspects of the manufacturing process, but are from a later stage than the main production period of the large statues.

**Table 5:** The Primary Sources About the Workshops Involved in the Production of the Statues and the Production Process Itself

Regest	Year	Short description
910	1508	Godl's brass workshop in Mühlau
924	1508	Kölderer designs the map of Mühlau (adjusted later)
949	1509	Peter Laiminger (Löffler) explains delays in casting
1100	1513	order of the creation of an inventory of the foundry
1137	1513	inventory of Sesselschreiber’s workshop
1250	1516	inventory of the work of Sesselschreiber
1322	1518	the emporer shows no more patience with Sesselschreiber
1326	1518	Sesselschreiber is dismissed and Godl hired
1339	1518	Godl casts Albert IV, Count of Habsburg (no. 20)
1344	1518	crucibles from Nuremberg
1772	1529	Magt complains about Godl
1957	1534	inventory of all statues so far cast
1989	1535	Kölderer's expertise on Sesselschreiber's statues
3010	1528	Godl's expertise on Sesselschreiber's statues
3011	1528	Kölderer's expertise on the castings so far
6746	1548	Gregor Löffler’s reports on the progress of his work
6850	1550	Gregor Löffler’s bill for the statue of Clovis
10307	1571	Lendenstraich notes the casting of the statues

#### Organisation of the Work

The casting of large amounts of liquid requires careful organisation of the labour in various process steps. This is true in today's foundries and even more so for foundries solely relying on manpower. Where the sculpting and moulding could be done in principle by a single person, with the occasional assistance of hands when moving heavy objects, this is impossible when melting and subsequent pouring of the liquid metal is to be undertaken.^24,25^ We have to imagine a larger undertaking with about 6 to 12 people involved.

Whereas the sources tell us that Sesselschreiber, as well as Godl, did employ various craftsmen (Table 6), the sources also tell us that Seeselschreiber did not always have the same amount of hands in his employ: sometime more, sometimes less, but in general he employed two wax carvers, two foundrymen, a black smith, a fettler and a painter (for instance Regest 1101).

**Table 6:** Indications on the Number of People Working in the Workshops of Godl and Sesselschreiber

Regest	Year	Artist	English translation of original German text
937	1509	Sesselschreiber	Gun founder Peter Laiminger (löffler)
938	1509	Sesselschreiber	Gun founder Peter Laiminger (Löffler)
970	1509	Sesselschreiber	Goldsmith for necklace
975	1509	Sesselschreiber	The Emperor has written to the government in Innsbruck, requesting craftsmen, cutters, and casters for Sesselschreiber's work on the statues.
1044	1511	Sesselschreiber	...if he could produce four pictures within a year. He replied positively, but indicated that he would require additional resources and labour.
1055	1511	Sesselschreiber	Goldsmith is paid for 4 weeks of work
1101	1513	Sesselschreiber	[Workshop expenses in 1510] He did not always have the same number of journeymen, sometimes fewer, sometimes more, but generally kept a painter, two carvers, two foundrymen, a fettler and a blacksmith.
1101	1513	Sesselschreiber	On the Sunday before Easter Sunday in 1513: Master Gilg Sesselschreiber's salary and his journeymen, as well as painter, cutter, caster, fettler, smith, and so on.
1102	1513	Sesselschreiber	Should the government, however, find that Sesselschreiber, as he claims, needs more servants, be they goldsmiths, stove-makers or others
1224	1516	Sesselschreiber	Son and so-in-law (Christof Seselschreiber and Sebastian Häuserer)
1245	1516	Sesselschreiber	Son and so-in-law (Christof Seselschreiber and Sebastian Häuserer)
1250	1516	Sesselschreiber	Son and so-in-law (Christof Seselschreiber and Sebastian Häuserer)
1253	1517	Sesselschreiber	The regiment instructs the chamberlain to pay Master Gilg, painter, 50 guilders for the dispatch of several of his servants, as painters and reapers, to whom Gilg claims to be indebted…
1256	1517	Sesselschreiber	Since he now found 50 Gulden necessary to pay the other servants, as carvers, painters and foundrymen
1449	1521	Godl	…with journeymen and others…
3010	1528	Godl	...together with my two journeymen, after the inspection.
1338	1518	Godl	Godl and his journeymen
1504	1524	Godl	Godl's brother Melchior
1583	1525	Godl	…also my journeymen
1594	1525	Godl	Godl receives 25 Gulden for five journeymen in his workshop in Mühlau
1775	1529	Godl	King Ferdinand takes Melchior Godl, brassworker, brother of Stefan Godl, as his servant.
1776	1529	Godl	Godl is also to be granted a home and workshop for himself and his journeymen as before. ...to carry out the work assigned to him with the utmost diligence to the best of his ability and to provide his workshop with good servants and disciples, but in particular to take on disciples who are from the county of Tyrol and to teach them the craft of *Rothschmiederei*.
1915	1532	Godl	My journeymen have nothing more to cut and work and now have to celebrate because I have no more *visierung*.

There were 16 crucible furnaces as well as 6 crucible tongs in the workshop. When a large mould is cast in any copper alloy the pouring of liquid metal must not be interrupted. With six crucible tongs, as well as enough hands to use them, a large statue can be cast comfortably without fear of cold shuts. It is reasonable to assume that the crucibles were pulled from the furnace in close sequence so as to allow uninterrupted casting.^25^ Even for 16 furnaces, six crucible tongs do not need to be in operation; three crucible tongs in operation should suffice. The crucibles are pulled from the furnace and then carried by another person, the founder, to the mould to be poured. In all likelihood this was not done with the crucible tong, but with another tool, which might be best described as a simple cradle in which the crucible is transported to the mould. These are not mentioned in the sources, so the foundrymen might have accomplished the task with the tongs instead. Without knowing the shape of the crucible tongs, this particular question must remain open.

The size of the Nuremberg crucibles remains unknown from the available sources, but clay-based crucibles rarely exceed sizes holding more than 20-30 kg, due to the clay qualities available. All the furnaces had to be monitored during the melting process. The operation of so many charcoal fired furnaces requires full-time supervision. They do need very regular fuel charging, if peak performance is desired. Sixteen furnaces cannot be operated by one person alone, but two furnaces per person is easily manageable. It is reasonable to assume that all sixteen furnaces can be managed by three to four dedicated hands.

#### The Manufacturing Process According to the Sources

Only a limited number of sources provide insight into the casting of the *Schwarze Mander*, as shown in Tables 4, 5 and 6. We will proceed to cite, discuss, and interpret the various sources in the sequence of the statue's production process. For all the processes, we only gain limited insight into certain stages of the production process. Unfortunately, the sources are mostly invoices and inventories rather than treatises on the craft. So the most pressing questions about the nature of the raw materials and their preparation, the exact process of moulding, the design of the gating system, and the melting and casting are sadly left in most part in the dark. There are, however, a few sources which help us to understand the process to some extent. These, together with a detailed technological study of the cast statues, should enable us to paint a complete picture of the manufacturing process. The following selection of sources will serve as a starting point for this endeavour.

#### Visierungen - Visualisation for the Sculptor

**Figure 4:**
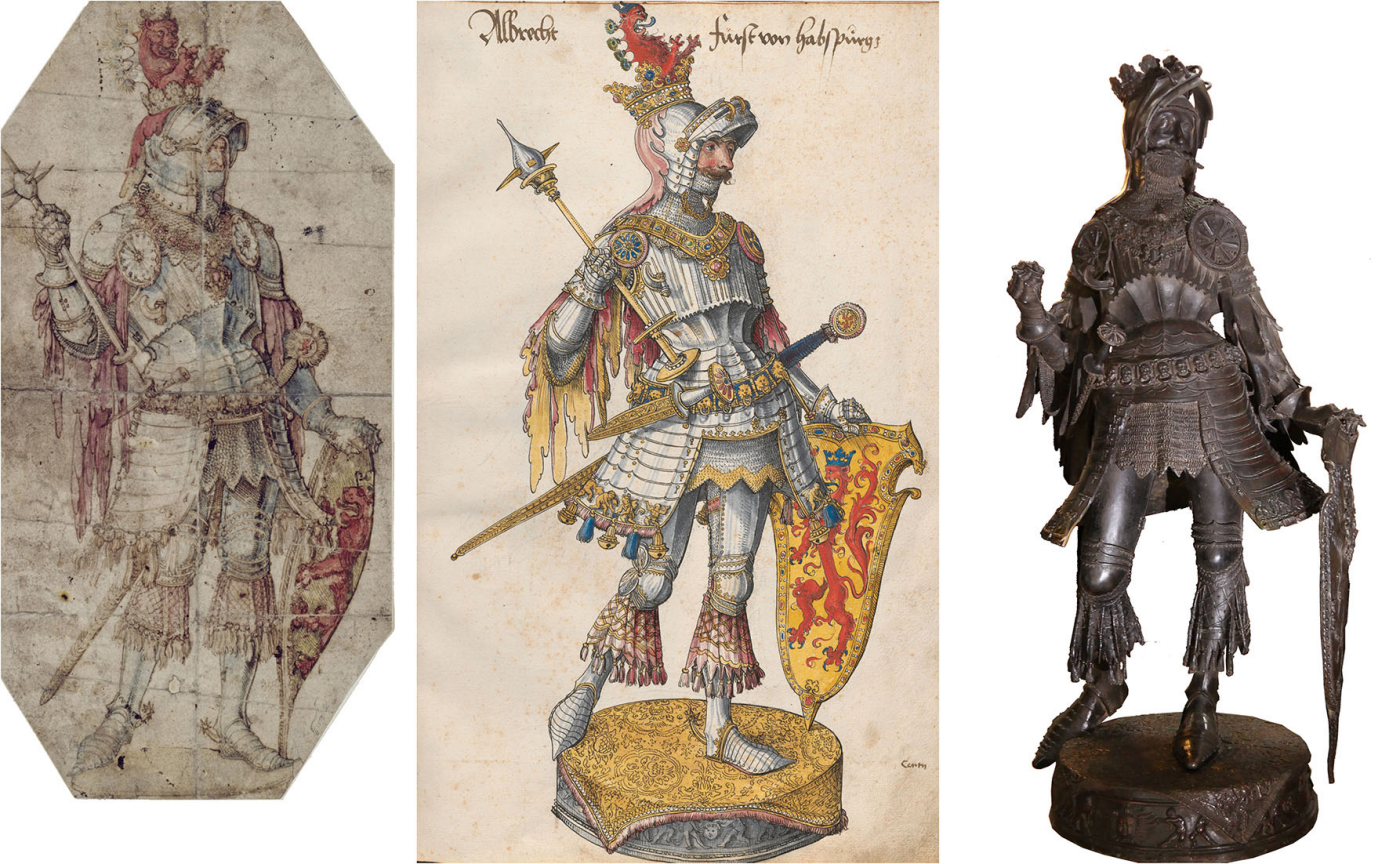
The statue of Albert IV, Duke of Austria (1298-1358). Left: Dürer’s draft from 1513/1514 (Staatliche Museen zu Berlin, Kupferstichkabinett / Jörg P. Anders; PDM 1.0 DEED; https://id.smb.museum/object/1043583); centre: Kölderer’s drawing from 1522^11^ (Wien, Österreichische Nationalbibliothek, Cod. 8329, fol. 15); the statue today, cast by Godl in 1518. Clearly Kölderer knew Dürer’s draft. Unfortunately, the visierung on cloth is not preserved.

The statues' initial designs, used as a guidance for the sculptor, were created on *tuch*, large pieces of cloth, presumably on canvas. It appears that these visualisations of paintings provided all details on the proportions, decorations and features in a scale of 1:1. These were called *visierungen*, and we will use this *terminus technicus* throughout this paper. In Sesselschreibers case these *visierungen* were made by himself since he was a painter and only self-taught in the art founding (Regest 934 and 937). A fact that ultimately caused a lot of pain for himself and the emperor. Table 2 presents all artists, including Kölderer and Polhaimer, who created the work drawings for the various statues (Regest 1844). In 1530, Hans Polhaimer (Polhammer) the older (painter) was paid for a design „*auf ain tuech nach Mühlau*" (a draft on a cloth/canvas), which probably refers to the visualisation and design for the last statues cast by Godl (nos. 1, 13, 17, 23-25).^8^ The drawings by Kölderer of the already cast and planned statues, specifically those made after the initial drafts of Sesselschreiber, are highly intriguing.^11^ It seems as Kölderer made drawings of the statues which were already cast, taking into account also the previous drafts and *visierungen* of Sesselschreiber and others (Figure 4). In the case of the statues cast after 1522, Kölderer probably used Sesselschreiber's earlier drawings (which had already been approved by Maximilian I) as a basis and may have slightly adapted them; his drawings later probably also served as a basis for the final *visierungen* for the casting, as also suggested,^10^ convincingly arguing that these were indeed quite precise working drawings in preparation for the sculptor's work.

Regest 1137 from 1513 lists the *visierung auf tüecher* of various statues. In 1532, Godl voiced his concern over the lack of *visierung*, which led to his journeymen being without work and ultimately resorting to partying (Regest 1915). In 1534, an inventory made after Godl's demise revealed 18 visualisations of the statues on large cloths (canvas), possibly on a 1:1 scale (Regest 1957):

“*...Mer achzechne gemalte visierungen der grabpilder auf grossen tüechern vnd acht vnd zwainzig gemalte schilt vnd drei gemalte tafln, …*

*“,*..more than eighteen painted drawings for the sepulchral effigies on large cloths and twenty-eight painted shields and three painted [wooden] panels, ...”

In some cases, we also find a payment in the form of cloth (*tuch*) for the casters (Regest 1338 from 1518; *tuch* for 50 fl for Godl). Although we have limited information on the size and design of the statues on cloth, the details are slightly clearer regarding the creation of models and moulds for casting.

#### Geschnitten von holz und wax - carved from wood and wax

The *visierung* assisted the sculptor in his task. The sculptors appear as *pild schnitzer,* literally “image carver”, or *wax schnitzer* [wax carver], both terms often shortened to *schnitzer* [carver] only. It cannot always be decided as to whether these artisans were wax carvers or wood carvers. This is important, because of a controversy of the attribution of carved wooden busts as casting models^7^ or more general models^10^ fulfilling a similar function as the *visierungen*, i.e., as an aid for the wax carver to produce the casting model from wax. The inventory of 1513 lists carved wooden busts and it needs to be addressed to which extent these are casting models or more general models (Regest 1137) (Table 3):

*“...Item fraw Margret, kais. mjt. tochter, ist von holz geschnitten vnd mit rupfein leinbat überzogen.*

*Item herzog Ernst von Osterreich ist geschnitten von holz vnd possiert.*

*Item ain hülzeins geschnitten prustpild, fraw Zira von der Mass, kais. mjt. muter…”*

...Item lady Margret, daughter of His Imperial Majesty, is carved from wood and covered with [rough, raw] linen cloth

Item Duke Ernest of Austria, is carved from wood and [posed, modelled, sculpted]

Item a wooden carved bust portrait, Lady Zira of Masovia, His Imperial Majesty's mother [sic!]...

The exact purpose of the wooden busts in this inventory is uncertain, but we can draw a few conclusions. Grammatically, it is unclear whether the statement that Margaret's bust is exclusively carved from wood is conclusive. The phrase could alternatively imply that this bust depicts Margaret and was created on a wooden substructure or armature, similar to how Ernest's bust was made. It is definite that this portrait is incomplete as the wooden object is covered in rough cloth or fabric. The terms "*rupfein*" and "*leinbat*" are interchangeable^26^ and probably refer to rough linen fabric.

Ernest, the Duke of Austria, is also made from wood and. “*possiert*” It is worth mentioning that the word "*geschnitten*" [carved] is not used to describe its production, which suggests that this model was not created as a casting model. However, the term "*possiert*" is only mentioned in five sources (Regest 974, 1137, 1610, 10226, 10307) and used in a more general sense. It can be more translated as “modelled,” “posed,” or “made to look like.” The material that was applied is not specified, but the bust appears to be already finished. The final wooden bust mentioned is that of Lady Zira (Cymburgis of Masovia), also made of wood.

The fabric covering Margaret's bust can be viewed as a transitional layer between the wood and a projected and theoretical wax layer, utilised to portray the individual being depicted. Oberhammer^10^ and Oettinger^7^ concur in this regard but differ on their intended purpose. Whilst Oberhammer considers these busts to be simply three-dimensional *visierungen* attached to a wooden core, Oettinger disputes this and proposes that these were finely finished wooden models, and that these were used to create the wax models by coating them with a layer of wax of the intended casting thickness. The cloth was utilised as a means of removing the wax layer. After removal, this wax would later serve as the casting model. Conversely, Oberhammer suggests that the wax carver created wax models directly onto casting cores made from loam. This, as we shall see below, contradicts the written sources and must also be rejected.

**Figure 5:**
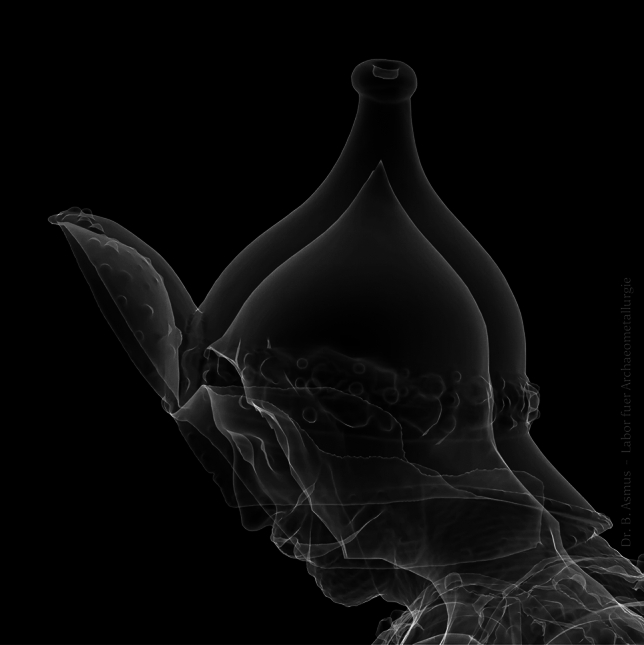
Based upon a 3D scan of Theoderich and the total weight of this statue, an average wall thickness was calculated. For visualisation purposes a hypothetical uniform layer was applied on the inside. 3D scans of the inside would certainly aid in the interpretation of how the models were made (Foto: B. Asmus).

Adding a layer of wax to a completed wooden model is impractical. The wall thickness of the cast black men is excessive. The average wall thicknesses of 2.5 to 3 cm (Figure 5) render this process implausible. What benefit does this process bring? Here, we have to take into account that the artists were paid 28 fl / cwt (Regest 1132; 3011). However, this did not escape the attention of the Innsbruck government, which informed Maximilian I that he would suffer losses whilst Sesselschreiber would benefit greatly by casting thicker (heavier) statues (Regest 1105). From Magt’s complaint about Godl (Regest 1772) we also know that the casting thickness varied widely.

The resulting wax model is disproportionate, and the finely cut wooden portrait remains concealed under the heavy layer of wax. Oettinger's hypothesis is not feasible in practice. However, with modifications and integration of Oberhammer's proposal that the busts were modelled on a wooden core, a more practical hypothesis arises.

If we accept Oberhammer's suggestion that the wooden parts of the portraits are only substructures, armatures, or false cores, then a layer of wax of suitable thickness would enable the wax carver to maintain the proportions of the portrayed person. This hypothesis could also explain the absence of preserved wooden models. A roughly hewn wooden core of a portrait was likely to have been disposed of, rather than kept. Examining the inner surfaces of the black figures is likely to provide valuable information about how the wax models were produced.

#### Furmen - Moulding

Regest 949 comprises a letter to Emperor Maximilian from Peter Laminger, explaining the reasons for the delays in casting a large bronze sculpture. Laminger had cast the initial pieces of the statue for Master Gilg Sesselschreiber prior to the latter's own workshop being established in 1511.

*„..vrsach halben die formen ob dem pild kan vnd mag ich pey dem fewr nit trucken; es muss von im selbs am luft trucknen, dan das pild ist als von wachs gemacht vnd wan ich das pey dem fewr wolt trucken, so zergieng das wachs vnd wer all arbait daran verlorn.“*

“..because the moulds of the sculpture I cannot and will not dry by the fire; it [the mould] must be left to dry by itself in the air, as the sculpture is made from wax, and if I were to dry it by the fire, the wax would melt and all work were lost.”

In this passage, we learn that the model is made from wax and the mould must dry naturally in ambient air. It is detrimental to the wax model to expedite the drying process near a fire as it might damage the wax. From this we can infer that the moulds mentioned in this passage are incomplete and possess exposed wax portions. There is further evidence for this interpretation in Magt’s letter of complaint (see below Regest 1772), where exposed wax portions are mentioned as well. If the outer loam mantle had been applied to the moulds already, a potential melting off the wax would have been acceptable, since de-waxing is the succeeding step of the moulding process. The reference here is to partially-finished moulds, meaning moulds that have received the loam core and require drying before being returned to the sculptor for the finalisation of the wax carving. This is confirmed in the subsequent section of the letter.

„*...dan ich muss in jedweders ainen besundern kern oder furm machen. Darnach antwurt ich im die Schenkel, arm vnd das pild zu dem leib, darnach so macht maister Gilg die geschmeid vnd clainoter darauf; über das mach ich den rechten auswendigen form.*“

“...then I have to make for each a special core or mould. Thereafter I return the legs , the arms and the torso to him, and Master Gilg proceeds to add the jewellery and gems; Then I proceed to make the outer mould by applying loam onto the models.”

Laiminger explained to the emperor that after receiving the models from Master Gilg [Sesselschreiber], he had to make a specific core or mould for each one. Afterwards, he returns them to Master Gilg who attaches the *geschmeid* and *clainoter* [jewellery and gems, or more generally decorations]. The finished models were returned to the founder who added the outer layers of loam to create the complete mould. The first source only provides information up until this point in the process.

There is an additional piece of information shared by Gregor Löffler in 1548 (Regest 6746) and Reisinger in 1574 (Regest 10521): Reisinger notes that the mould cannot be made in winter, because it does not dry by itself in the cold conditions; Löffler notes that such moulds have to dry in a warm room with constant temperature:

“...so last sich doch dise arbait mit nichten eilen sonder muess ir weil und zeit haben, soll ich änderst die arbait rain, sauber und zierlich vertigen; dann es muess der form solchs pilds fein, senfts, stetigs mit ainer warmen Stuben getrucknet werden.”

“...so this work cannot be done in a hurry, but must be given time and patience if I am to produce the work well, properly and neatly. Because the mould of such a sculpture must be dried carefully, gently and steadily in a warm room.”

In 1516, the next source is yet another inventory (Regest 1250) ordered by Maximilian I, because of the slow progress of Gilg Sesselschreiber. It lists all the works carried out so far, but most important for the reconstruction of the process is a section on the moulds for the statue of *Fraw of the Mas,* Cymburgis of Masovia (no. 5):

*"ligt noch der form beim ofen, hat seider das wachs daraus gelassen und zu stucken erlegt, ist fertig bis zum guss."*

mould still in front of the furnace, it is since drained from wax, is separated into several moulds, ready for casting

The next source directly related to the creation of the sculptures dates to 1529. A lot did change in the two decades since the initial source: In 1518 Gilg Sesselschreiber was dismissed from his position due to his below-par performance. Stefan Godl, another founder from Mühlau who had been in the service of the emperor since 1508, was appointed in his place. The report, written by Leonard Magt and addressed to Jörg Kölderer, the administrator and architect of the emperor's tomb, highlights deficiencies in Stefan Godls' work. We cannot determine the accuracy of these reported shortcomings or whether Magt had an ulterior motive. Nevertheless, the report provides insight into the manufacturing process; the important passages are discussed below (Regest 1772 from 1529):

"..die Zubereitung des Lehms dann „*in ainer wochen*" erledigen wolle, ,,*daran mir etwanen 5 oder 6 wochen zuberaitt sollten haben' \ daß deshalb der Lehm „grien vom perg her*" gebraucht werden müsse, wodurch die Form „*grob vnd etwas unrain*" werde."

“The preparation of the clay would then have to be done in a week, for which we normally need 5-6 weeks and that therefore the clay would have to be used “green from the mountain,” and therefore the mould would become coarse and somewhat impure.”

The opening section explains that the moulding loam preparation process should take up to 5-6 weeks to be done correctly. If, however, the preparation is done incorrectly, as Stefan Godl apparently did, the surface reproduction of the mould material will be inferior. Regrettably, we don't learn much about the composition of the loam, except that it comes from fresh sources in the mountains and may contain some fresh loam pit material. This badly prepared moulding loam led to rough and slightly flawed moulds. The preparation at least included a sifting step. The inventory of 1513 (Table 3) mentions an iron sieve used for this purpose, which supports this conclusion (Regest 1137):

“*Item ain eisne reyter zum sand vnd laym*.”

“Item one iron sieve for sand and loam”

As laid out above the preparation possibly included a fermentation process as well, in which the organic matter within the loam was further broken down. The inventory mentions a tamping mill for “*rotten laym*.” Only towards the end of the casting of the black men one of the last foundrymen, the gun founder Lendenstraich, offers another glimpse into the loam preparation as well as on the casting process (Regest 10307):

“..*denn die lämbgrueben, darinnen man die pilder geusst, muess wider mit erd ausgefüllt werden*.”

“...because the casting pit, in which the statues are cast, has to be filled again with earth.”

Moreover, he needs 2000 bricks, iron wire, wool clippings and calves hair to be mixed with the loam. These are typical ingredients of moulding loams and provide organic temper, mostly responsible for additional plasticity, to facilitate drying and most importantly to increase the gas permeability of the finished mould. The large amount of bricks may have been used to build a temporary kiln to bake the moulds. This is not recorded in the sources, but Theophilus Presbyter advises such a procedure already in the 12th century when baking bell moulds.^19^

The following passage focuses on the moulding process itself. The moulds in the workshop were frequently left incomplete for extended periods. Magt provides a report on this (Regest 1772):

*"...halbe Theil geformbt, der ander theil im wax ist vnd also ein zeit lang steen muess, so zerfeilt des wexen vnd wird vil daran erprochen, des ich darnach von nuiem wider machen mues, vnd wird so rain nymer als es vor gewesen ist.*"

“..half the part is moulded, the other part is in wax and it does therefore stand for a while, the wax is suffering damage and deterioration. so that I have to repair and redo it again, and it will never be as neat as it was before.”

Only a part of the model is moulded, whilst the other part of the wax remains uncovered. Leaving the wax exposed for long periods of time can damage the model, requiring it to be remade. However, it will not be as pristine as its original state. There are also some things we can learn from this. The wax model is formed in a series of steps. During the process, the wax model is left uncovered for those periods, in which the loam must be allowed to dry. Both 1513 and 1516 inventories refer to moulds in an unfinished state. Unless the moulds sustain mechanical or thermal damage, there appears to be no inherent harm resulting from the long drying process. The third extract from Magt's report is the most controversial one, because of the ambiguous terminology he uses for this crucial step in the mould making:

"..*.vnd rait* [berechnet] *nit vor hinaus, wie er die stuck vnd die formen von dem kern pringen wöll, daraus aber nachtail kumbt. Wann so darnach der kern gemacht wird in die form vnd so mans wider von ainander tuet vnd abzuicht, so prechen denn stuck aus dem kern, desgleichen auch oft aus der form, dardurch die güssdicke ungleich an dem pild wird vnd oft an einem ort vil dicker wann an dem andern, darvon die überig schwer kumbt*. "

“...and does not consider how to remove the pieces and moulds [or models] from the core [armature], which results in complications. When the [casting] core is subsequently made into the mould [rather: model] and dismantled, fragments often break away from both the core and the mould. This irregularity causes uneven casting thickness, often resulting in a considerable thickness disparity in different areas. This is the reason for the excessive weight.”

From the above source it becomes clear that at least the terms *kern* and *furm* have a broader meaning than their modern counterparts core and mould. The word *furm possibly* relates to model as well as to mould, and the word *kern* in the first instance is not a casting core, but a core, i.e. an internal structure to facilitate wax carving. The sculptor carves the wax model, possibly in its entirety, to ensure proper proportions. The foundry master proceeds to divide the wax model into manageable pieces (*stucke*) and prepares them, by adding the casting core (*kern*) as well as the outer mould mantle.

#### Ausberaiten - Fettling

Ultimately, the sources are too sketchy to paint a coherent picture of the casting process, and the first step must be detailed analysis of the cast statues. Only by a meticulous study of the inner and outer surface of the statues may we arrive at a viable process hypothesis. However, what we can observe from the letter of complaint from Magt is that the division between the founder and sculptor is still active. Stefan Godl, the founder, is in control, and the wax carver works under his supervision. The wax carver's duty is to create wax models from the working drawings provided by Kölderer, Sesselschreiber, and others.

Since we do not know the details of the casting process, the only step in the process that is known to some extent is the cleaning and finishing of the cast statues. Again, there are mostly inventories from which we have to glean scraps of information. In general, fettling includes all stages after casting, i.e,:

1. The removal of the mould materials, specifically the removal of the casting core;
2. The removal of the gating system;
3. Closure all openings caused by the casting process;
4. Repair of all casting defects;
5. Joining of separately cast pieces;
6. Surface finishing, such as cleaning, chiselling, filing, scraping or polishing.

For the most part we do not learn much, but the sources repeatedly report that the cores had to be removed before weighing (such as Regest 1339, 1932), because the foundryman's salary was paid by the hundredweight of the finished object. Sesselschreiber and Godl's salary was 28 fl/cwt (Regest 1250, 1339, 1990).

Amongst the earliest is a note by Sesselschreiber that he employed a goldsmith to finish the necklace for Ferdinand of Portugal (no. 10) (Regest 1052 and 1101), as a brass fettler was not equipped to do this work. This particular statue is indeed the most elaborate and finest of the 28 statues. This changes considerably with his later statues, which are less fettled. This is true of all the workshops involved, including those of Sesselschreiber, Godl and Vischer.

Godl, who complains about the casting quality of Sesselschreiber’s statues in 1528 (Regest 3010), cites the need for extensive remedial work and additional metal, including the casting of new parts, forming new wax models, fettling, and the correction of casting errors. A good example is the statue of Cymburgis (no. 5):

*Das pild die von der Mass ist mer dann halb auszuberaiten und die löcher zu vergiessen; wirdet wol zu verprauchen 1 centner*

“The image of Lady Mass is still to be fettled for more than the half and the wholes need to be closed by casting on, it will require one hundredweight.”

This observation is confirmed by Kölderer in the same year (Regest 3011):

*...sein die luminiern an klaidern ubel gefallen und ist auch löcherig, …*

“...the decorations on the dress turned out badly worn and it is also full of holes.”

Another important point is the joining of the different parts of the statues. In the written sources, we can deduce both mechanical joining, usually with screws and/or iron bars on the inside, or cast on (as the base; Regest 1957). Concerning the latter, we are informed which statues were screwed or cast on their base. For some statues, iron nails, screws and bars were noted to be placed on the inside in order to join some parts together (Regest 3011: Kölderer reporting on Sesselschreiber’s work on the statues no. 10, 14, and 15):

*Ruedolff, Römischer kunig, grave zu Habspurg und Kyburg: ist gössen von stuckhen, halten's eisennagl und schraufen auf einander verfestnet, …*

*Kunig Ferdinanndus zu Portigall: ist gössen; manglt am halspant zwen trackhen, zwai stuckh; und ist das pild von stuckhen gössen, sein grosz eisne Stangen darinne, mags inwendig nit sehen, wie es gestalt ist.*

*Philips, kunig zu Hispania, erzherzog zu Österreich, herzog zu Burgundi etc.: ist gössen von messing und von stuckhen, steet auch auf eisnen Stangen in der bruest entzwai*

Rudolph, Roman King, Count of Habsburg and Kyburg, is cast in pieces, held together by iron nails and screws

King Ferdinand of Portugal: is cast, is missing two dragons on his necklace; the sculpture is cast in pieces and has a large iron bars inside: I cannot look inside, so I cannot tell exactly how it is held together.

Philip, king of Spain, Archduke of Austria, Duke of Burgundy etc., is cast in pieces from brass; is also held up by iron bars in the chest.”

Unfortunately, no further information has been found in the original sources regarding the joining of the single pieces of the statues.

#### Vergulden - Gilding

The emperor originally wanted the statues to be gilded.^5^ In 1511, Sesselschreiber requested and received gold, presumably for fire-gilding the statues to be executed by his hired goldsmith (Regest 1052). In 1513, the government wanted to know the costs for gilding or silvering of the statues. As noted by Sesselschreiber, the cost of gilding would be approximately 500 fl per statue, leading to the exclusion of gilding and silvering for the time being (Regest 1108). In 1517, Sesselschreiber wrote about gilding 21 statues (Regest 1256), but never actually made it.

After 1513 and before 1520,^7^ Sesselschreiber complained that he had to cast some statues in copper instead of brass. For these castings he had to build a new furnace with four bellows, all at his own expense and for which he asked to be reimbursed. This furnace would then allow him to cast the copper statues, according to Sesselschreiber:

“*...in das mir unrecht geschicht, wirt auch euer g(naden) in demselben verdingbrieff finden, das mir die ganz arbait ist von mesing angedingt und nit von küpfer, mich hauskamer darin gedrungen von küpfer zů geůsen, ich die werchstat hab můssen verendern und ain paů tün üff min selbs kostung, ain gůsoffen zů dem küpfer gemacht und an das waser gericht mit IIII plasbalgen, solicher offen gestett mich mit siner zügeherung mer dan I*^*C*^* güldin*.”

“ ..in which injustice is done to me, his majesty will find in the same contract, that the complete work was commissioned in brass and not in copper, however the finance chamber forced me to cast in copper. I had to modify the workshop at my own expense, to have build a furnace for melting copper which needed to be driven by water power[ed bellows]. Such a furnace with all its accessories more than 100 Gulden.”

The new furnace was likely needed to achieve higher casting temperatures to melt the copper, which is more effective for (fire)gilding than brass.^27^ However, melting copper requires higher temperatures (copper melts at 1083°C) than melting brass (brass with around 20 wt% Zn melts at 1000°C).

Parts of the statue of Ferdinand I of Portugal (no. 10) were allegedly gilded after 1528, according to the inventory of 1534 (Regest 1957). Regarding the statue of Elisabeth of Carinthia (no. 27), Godl and Kölderer note in 1528 that the surface of the statue is smooth and polished; if it was intended to be gilded, the surface would have to be reworked (Regest 3010 and 3011). Kölderer notes in his report from 1528 (Regest 3011) on the statue of Elisabeth (no. 27):

“*Will ewr maj. ain guldin stuckh haben, muesz man's von newem mit stempflen aushawen und graben.*”

“If your majesty wishes for a golden piece, it has to be made again by punching, chiselling and engraving.”

The “*stempflen*” are most likely the punches used by the fettler or gold smith to work the surface in a chipless manner, the “*aushawen*” is the chiselling work, the “*graben*,” maybe translated more generally as engraving, which can be done by both, chisel and burin.

Concerning Mary of Burgundy (no. 26), Kölderer notes in the same place “*ist wol und sauber gössen mit aller zugehörung, von messing mit guldin stuckhen*” (well and clean cast with all details, of brass with golden pieces). The Regest 1250 (from the year 1516) notes that also the statue of Theodobertus, today not any more preserved and likely remelted, was also made of copper and was ready for being gilded:

*„Daran ist der leyb von kupfer gössen vnd der merer tail daran zum vergulden ausberait. Ligt wieuor, allein etwaz wenig zum vergulden ausberait vnd das haubt stet in formen.“*

“On that the body is cast from copper and the greater part has been fettled and prepared for gilding. It lies, as before, with very little fettling and preparation for gilding, and the head is still in moulds”

Likely, at the end only the statues made of copper (nos. 5, 14, 27 and 28) were intended to be gilded. Those statues were the last from the Sesselschreiber workshop and made between 1516 and 1518.

In the end, none of the statues were actually gilded; only traces of mercury as indication for fire gilding were detected recently on the swords of statues nos. 1 and 4, and are presented in this paper (see below).

#### Preliminary Process Description Based Upon the Written Sources

To summarise the above observations, we present a preliminary hypothesis on the casting process. We acknowledge that this process may have undergone changes throughout the extended period of creating all the effigies. Therefore, we must rely on the factual evidence available at this time. However, we were able to present new information by reassessing the primary sources and the secondary literature on the matter. The production process as it is recorded in the sources was presented above, our hypothesis is as follows:

1. Drafts by Sesselschreiber and Dürer, later also by others such as Kölderer, Polhaimer and Amberger;
2. *Visierung* that detailed out the sculpture, possibly in scale 1:1;
3. The wood carver produces a false wooden core;
4. The wax carver makes a wax model on the false core;
5. The founder makes the casting core into the wax model, and lets it dry by itself;
6. The wax carver works the wax model to its final stage;
7. The founder applies the outer layers of the mould and lets it dry naturally;
8. The wax is drained from the mould, but it is not recorded how this was done.

There is no information provided on the moulds' baking process, and no detailed information on the casting apart from the note that the loam moulds of the statues were cast in a casting pit in the ground (Regest 10307).

It is certain that all statues from Mühlau were made directly in wax. There is evidence for the limited use of wooden matrices for the production of surface ornamentation, however there is no evidence for mechanical copying of complete models in the modern casting sense. The latter emerged only from 1565 onwards when Alexander Colin began using plaster moulds for the mechanical production of wax models based on his original models (Regest 9712).

This short list highlights that crucial steps in the manufacture of these art pieces are still essentially unknown, mainly those concerned with the actual production of the actual moulds, the melting and the casting technology. On a more positive note it is reasonable to assume that several of these questions may be answered by a thorough technological investigation of the statues themselves. Most casting or moulding processes leave behind physical evidence that can be identified and are waiting to be recorded. An investigation of remaining casting cores, inner and outer surfaces, and not least the chemical analysis of the alloy composition of every single part of the statues will lead to a much more comprehensive understanding of how these statues were made.

## Chemical Analyses

### Methodology

Chemical analyses were performed utilising an Oxford Instruments portable Energy Dispersive X-ray Fluorescence (EDXRF) analyzer, specifically the X-MET5100 model, which is equipped with a high-resolution detector and a 45 kV Rh target X-ray tube (with a maximum of 50uA). The X-ray beam enabled spot measurements with an approximate diameter of 9 mm. The primary elements, including Cu, Sn, Zn, and Pb, were quantitatively detected, whilst other elements such as Ag, Pd, As, and Fe were only identified qualitatively, mainly due to the influence of corroded layers. Notably, S (sulphur) is hard to be detected by this particular ED-XRF setup.This is due to the fact that the peaks of Pb-M lines overlap with the S-K lines and are hard to distinguish.^28^ To ensure consistency, measurements were taken using the standard setup, involving a voltage of 40 kV, a current of 10 μA, and an acquisition time of 60 seconds. For calibration purposes, alloys similar to the ones of the statues were used as reference standards.^29^

Based on the quantity of the data a multivariate approach was followed following previous works^29,30^ to gain a deeper understanding of the challenges and information arising from a significant volume of interconnected data. Principal Component Analysis (PCA) is a valuable method that employs a multivariate approach to extract the most information from acquired data. It's important to note that variables are not necessarily independent from one another, and by considering them in isolation, we may overlook significant information. By integrating all available information, we can uncover even more relevant insights from the gathered data. PCA conducts a transformation of the initial data space, identifying new directions as the plane of maximum variance, carrying most of the information. The data set X_o,v_, with o rows and v columns, is then decomposed into a score matrix S_o,c_, containing o rows and c columns (where c represents the number of key components), a loading matrix L_c,v_, comprising c rows and v columns, along with an error matrix E_o,v._. In this study, X_o,v_ represents the initial dataset, S_o,c_ serves as the score matrix providing insights into the relationships amongst the designated samples (single ED-XRF analysis), and L_c,v_ acts as the loading matrix revealing the correlations amongst the variables (elements detected). The data set was analysed with an R- based chemometric software, CAT (Chemometric Agile Tool) software.^31^

### Results and Discussion

About ten analyses were conducted per statue using a portable XRF instrument. It is worth noting that these analyses solely offer initial insights into the chemical composition of some, but not all, of the different parts of each statue, as it is still unknown how many different pieces each statue was made of. Therefore, it is crucial to examine each statue internally and externally to gain more knowledge about the nature, shape, and quantity of individual cast parts.

Most of the statues were made of brass, usually with less than 3 wt% of Pb and less than 2 wt% of Sn. In the few cases where higher quantities of Pb or Sn were measured, these were mainly associated with separately added items such as a crown, sword or dagger, but also with the face and hands of some of the statues. This could either be related to technological or aesthetic choices (as discussed further down); the 'polishing' with Pb-Sn alloy, as was also found on the 12th-century bronze door from Gniezno, Poland, is in this case excluded (yet unpublished research during a currently ongoing research project of one of the authors (M.M.)). Some measurements indicated a relatively high iron content (up to 4.7 wt%) in certain areas of particular statues. As this was not consistently observed, contamination from the environment, artificial corrosion, joinage residues or core pins might be the most likely reason.

Elements such as Ni, As, Sb and Ag can indicate the type of copper mineral used to produce the copper, such as Fahlore or chalcopyrite.^28,32^ Considering the standard deviation, measurement conditions, and instrument limits, and the fact that the measurements were taken from the corroded surface of the statues, only the presence or absence of these four elements are deemed significant for a potential discrimination of the copper used by the various artists. Arsenic is detected, ranging from 0.1 to 0.5 wt% in all statues except for Nos 2 and 19 (Godl) and no. 26 (Sesselschreiber). Nickel is only present in the statues created by Godl, Löffler, and Vischer, with a range of 0.1–0.3 wt%. In the case of the Sesselschreiber statues, only three external parts showed the presence of 0.1 wt% Ni (no. 11: chain and sword; no. 14, sword), at least with our current XRF measurements. Having a look at the AAS-analyses carried out by J. Riederer in the earlier 1980s,^12^ one notes that Ni is anyway present in all the statues, even though with considerably low amounts, and significantly lower amounts for all the Sesselschreiber statues. Whilst Ag is regularly present in Sesselschreiber's statues, only four of Godl's statues (nos. 4, 18, 19, 21) had some Ag content. Silver was also detected in one of Vischer's statues (no. 12) and in the last statue cast, no. 16, which was created by Löffler. Small amounts of Sb (0.1–0.5 wt%) were found in over half of the statues. The presence of larger quantities of Sb (0.2–0.5 wt%) is distinctly linked to the presence of Sn, and is primarily evident in added components of the statues such as swords, shields, and other similar items, as well as the face and hands. This is interesting insofar as we know that Godl and Sesselschreiber received copper from the same mine, the “Tauferer copper” - which obviously did not contain copper with the same chemical composition.

Four statues were made of copper (statues nos. 5, 14, 27 and 28), likely because it was planned to gild them (see above). It should be noted that no traces of gold or mercury were found on the surface of these statues in the XRF analyses carried out in this study. However, small amounts of mercury were found on the swords of the statues nos. 1 and 4: this might be an indication of local fire gilding. The inventory of 1534 notes (partial?) gilding of the statue of Ferdinand I of Portugal (no. 10) after 1528 (Regest 1957). However, also on this statue no traces of gold or mercury were found in the XRF analyses carried out in this study.

**Figure 6:**
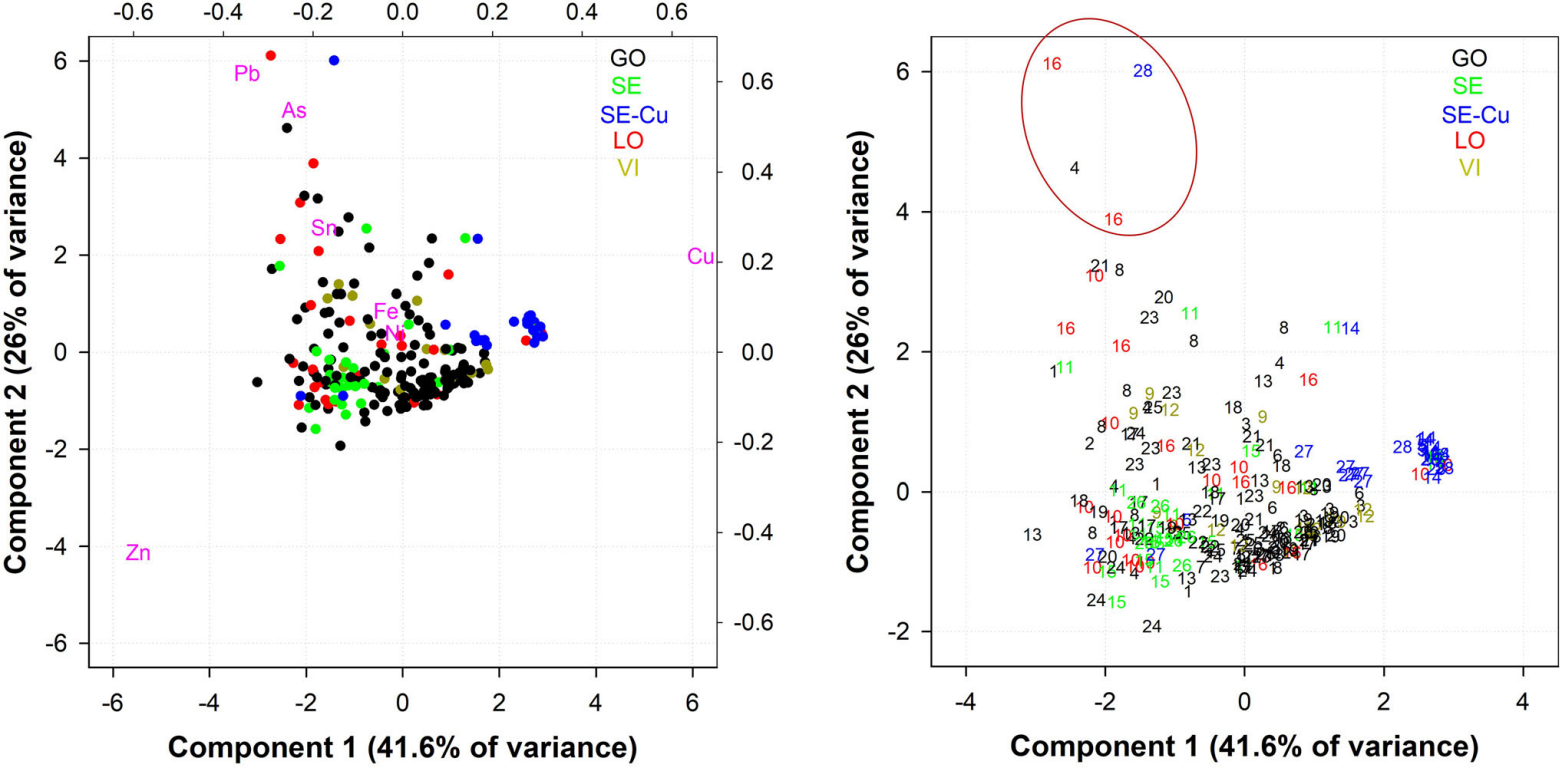
PCA data elaboration. Left: Biplot of the ED-XRF raw data. Right: Score plot with ED-XRF measurements labelled according to the number of the statue and the artist: GO= Godl; SE= Sesselschreiber; LO= Löffler; VI= Vischer.

For the PCA, the whole set of data was arranged in a matrix of 249 rows and 7 columns corresponding to the measured points of ED-XRF and the elements composition, respectively. We decided to exclude Sb from the PCA, due to null variability, which would have precluded proper processing of the data. A preliminary screening involving all raw data is displayed in Figure 6, where a biplot and a score plot of the first two components are presented (PC1 vs PC2, total explained variance of 66.6%). The graph presents both the position of the variables (elements) and the samples (ED-XRF analyses) in the newly rotated space allowing for an evaluation of possible correlations between the elements and grouping or clustering of the ED-XRF points of analyses, based on their composition. Furthermore, each point (and each statue) was identified with a colour associated with a specific artist.

**Figure 7:**
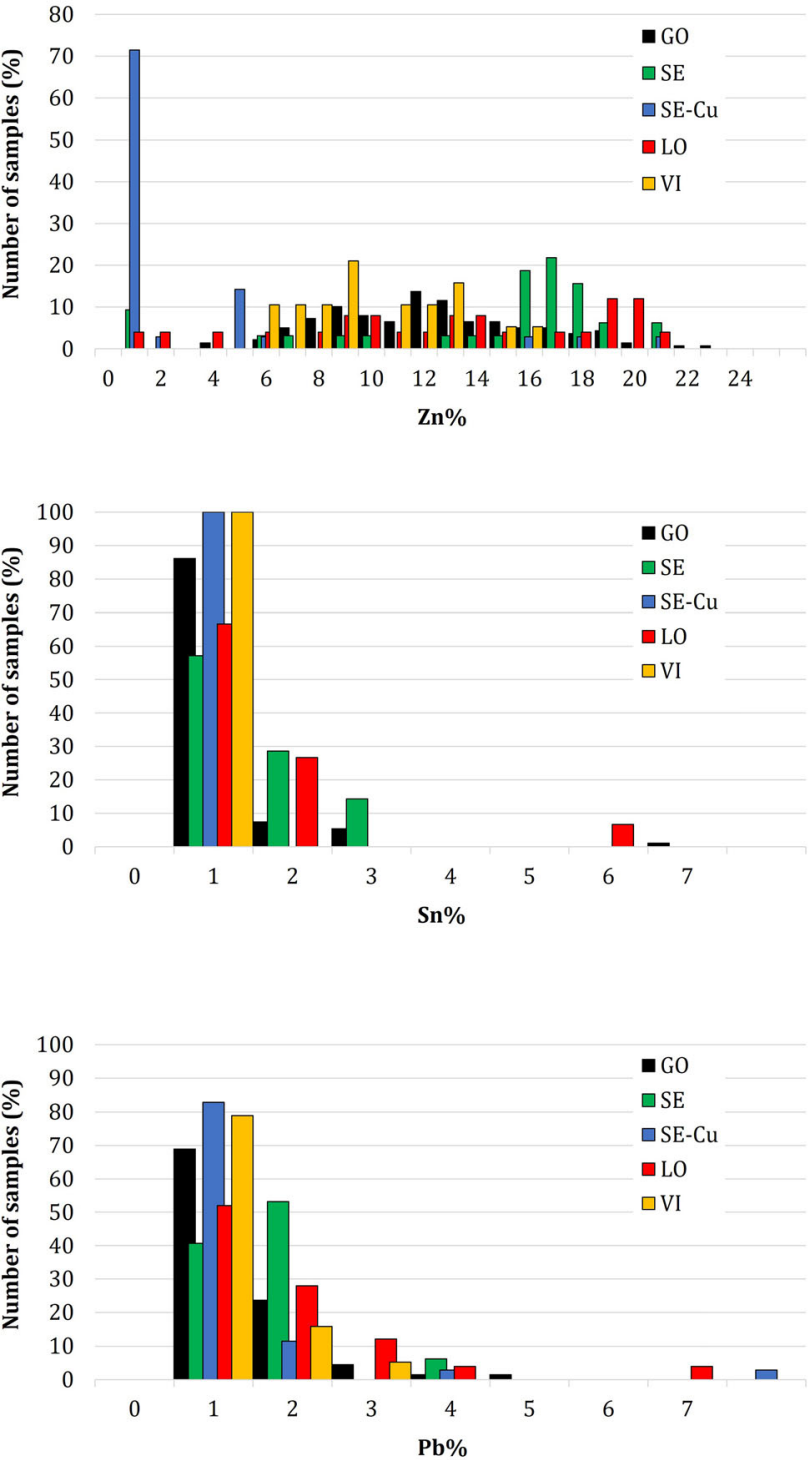
Frequency distribution of the main alloying elements according to the workshop categorization: (**a**) Sn; (**b**) Zinc; (**c**) Pb.

Most of the samples are distributed along the imaginary line that connects Cu with Zn, confirming that the alloys are made for the most part of **brass** with variable amounts of Zn. In general, it is not possible to distinguish a particular composition, nor can a single alloy be associated with a particular workshop or with a particular set of statues. In addition, some parts of some of the statues are made of a quaternary alloy (Cu-Zn-Sn-Pb), as can be seen by some points distributed towards the upper left quadrant of the plot, closer to the Sn and Pb variables. If we look at each workshop in isolation, we can make some observations. The statues produced by Godl (black dots) present a wide range of Zn content, also due to the higher statuary production (17 statues), with zinc varying from 3.5 to 22 wt% and variable Sn and Pb content (Figures 6, left; 7). Sesselschreiber clearly chooses two different types of alloys for its statues: one basically without Zn (average < 1 wt% of Zn, blue dots, a total of 4 statues) and one with an average Zn content around 17 wt% (green dots, 3 statues), with some parts richer in Sn and Pb (Figure 7). Löffler (red dots) and Vischer (yellow dots) use a brass with varying amounts of Zn (from 4 to 22 wt% and from 7 to 22 wt%, respectively, Figure 7), which is also "contaminated" with other alloying elements such as Fe, Pb and Sn. Interestingly, statue no. 16, cast by Löffler’s son Gregor in 1550, is in the upper left quadrant of the graph, indicating a higher average Sn content than the others, which is most likely related to the raw material used: old hackbutts and handgonnes, which obviously also contained some Sn (Regest 6783, 6787 and 6850). It is also interesting to observe that some ED-XRF measurements (circled in red, Figure 6, right) and parts of statues produced by Godl, Sesselschreiber and Löffler are located very far from the main “cloud.” These points were taken from statues 4 (measurement point 04_5, face), 16 (points 16_4, hand and 16_5, lower arm) and 28 (point 28_1, dress), and related to separately added items. However, the difference in composition to the other parts of the statue is unclear: as discussed above, the high content of Pb and Sn in the alloy could be the result of a deliberate choice to aesthetically alter the colour of certain parts of the statues (such as the face of statue 4 or the hand of statue 16) or a possible subsequent repair (dress of statue 28). On the other hand, the position of point 16_5 in the graph, related to a high Fe content (2.3 wt%), is most likely the result of contamination from artificial corrosion, joining residues, core pins or the presence of copper sulphides, or a combination of all of them. Concerning copper sulphides, it is not uncommon to find mixed sulphides of iron and copper (Cu_x_Fe_1-x_S_2_) in the matrix of copper alloys produced by direct smelting in the 16th century. Consequently, we cannot rule out the possibility that the presence of iron (also) comes from the ore mineral in the form of sulphides, which cannot be detected by the instrument used.^28^

**Figure 8:**
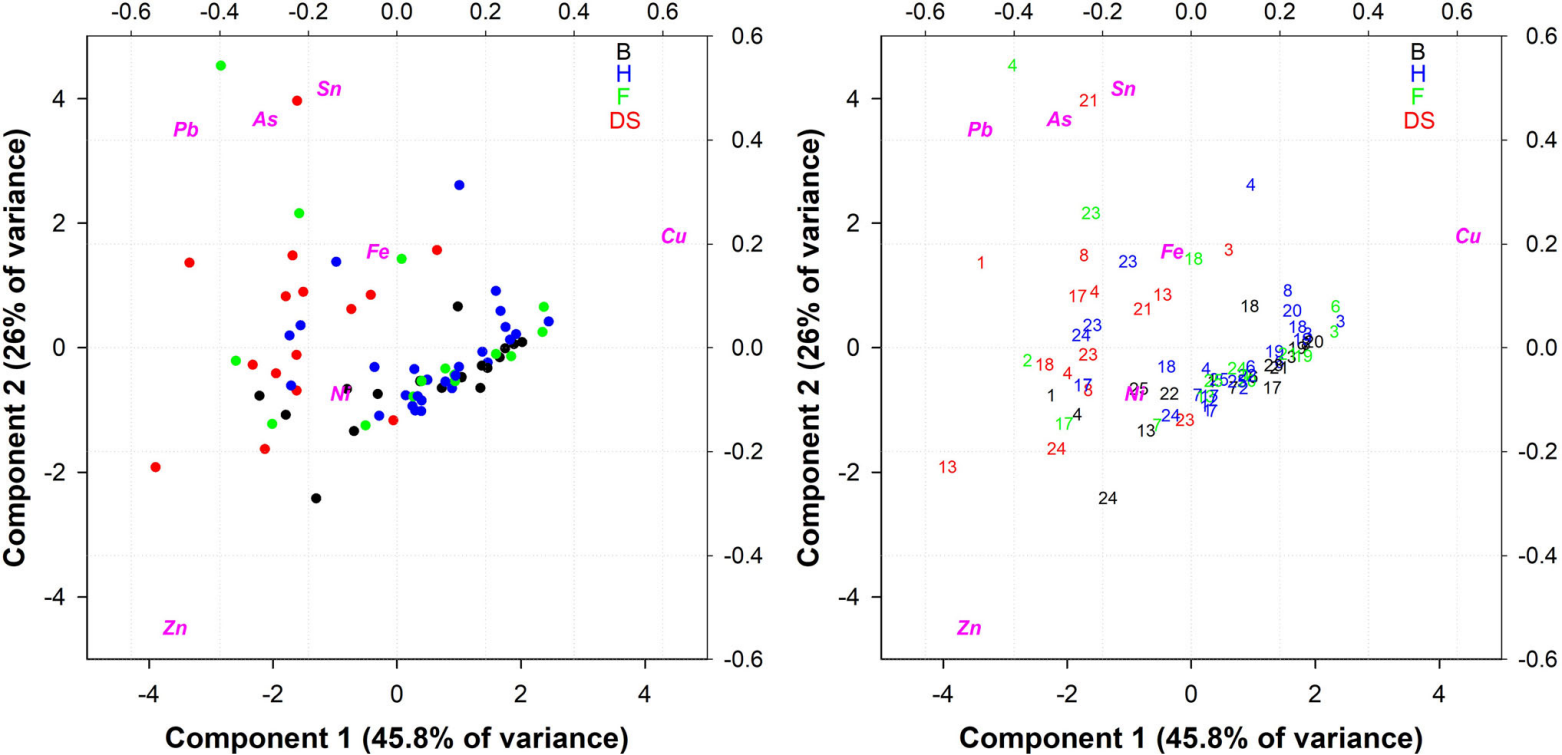
PCA data elaboration on Godl statues labelled according to the number of the statue and the statue parts: B = Base; F = Face or cheek; DS = Dagger or sword; H = Hands. Left: Biplot of the ED-XRF raw data. Right: Biplot plot with statue number.

Whilst analysing the PCA raw data, it was observed that the majority of the evaluated sections of each statue, including torso, feet, coat, and arms, exhibit a uniform composition. However, some individually attached pieces display a varied chemical composition. As such, two additional PCA analyses were conducted on single artists (Godl and Sesselschreiber) in order to identify potential differences in separately added objects such as dagger or sword (DS), the base (B), or freely visible body parts such as hands (H) or face (F). Figure 8 shows the PCA elaboration of the statues from the Godl workshop (biplot with dots on the left and with the statue number on the right). The plots show the first two principal components (PC1 vs. PC2), which together explain 45.8% of the total variance.

The wide range of data on the graph in this PCA confirms Godl's mastering of different alloys in the production of the *Schwarze Mander*. The same information can be obtained from the graph as previously mentioned - the alloy consists of brass with a varying composition of Zn, with certain parts containing additions of Sn and Pb. By examining the chart, it is evident that significant variations exist amongst the components of the statues. The base (black dots), the majority of the faces (green dots), and the majority of the hands (blue dots) exhibit conformity with the principal Cu-Zn line. In contrast, individual parts of the statues, including swords and daggers (DS), exhibit higher levels of Sn and Pb, as well as lower levels of Zn, in comparison to the other components. A few points are present in the upper part of the graph, showing higher Pb and Sn contents. These points include the face of statue no. 4 (measurement point 04_5) and the sword of statue no. 21 (21_3). Although they could be considered statistically anomalous, they have been included in the compositional variability due to the reliable chemical results, which are not influenced by surface contamination or instrumental errors. The sword of statue no. 1 (point 01_3) and the scabbard of statue no. 13 (13_6) have anomalous amounts of Fe above 1.4 wt%, which could be due to corrosion contamination. These two measurements are the only outliers in the left part of the graph.

In general, it can be seen that:

1. The base of all the statues (black dots) is made of brass with a chemical variability of zinc ranging from 6 to 20 wt% and a content of lead and tin of less than 1 wt%. Statues 1 and 4 are exceptions, with the average lead content at around 2 wt%.
2. Daggers and swords (red dots) display a more complex composition, characteristic of a quaternary copper-zinc-based alloy with high lead and tin contents. This complexity is further reflected in the shift from the copper-zinc direction towards tin and lead variables.
3. The faces and hands are made of either brass or a quaternary Cu-based alloy, where the Zn content is related to the alloy composition used to make the statue, and the Pb and Sn contents vary.

The majority of statues demonstrate a consistent content of Zn. However, a small number of statues have different alloys either in their hands or in their faces, or both. Likely, it was a conscious decision based on either technological (such as castability) or aesthetical choices (to represent the visible naked body parts are more natural colour). Based on such discrepancies of composition, we can classify these statues into three separate groups.

1. The first group comprises statues with a uniform composition despite having separately added pieces (statues nos. 1, 3, 7, 13, 19, 20, 21, and 22).
2. Statues depicting hands and faces with a higher Pb content of up to 2 wt%, despite the consistency of Zn content, can be found in statues no. 2, 17, 18, and 23 and the base of statue no. 11.
3. In contrast, statues 4, 6, and 8 exhibit hands and faces with a higher lead content of up to 5 wt% and tin content of up to 3 wt% and lower zinc content.

Regarding the other statues, no sufficient data on the chemical composition of face and hands are available (statues with hand protection also had to be excluded).

**Figure 9:**
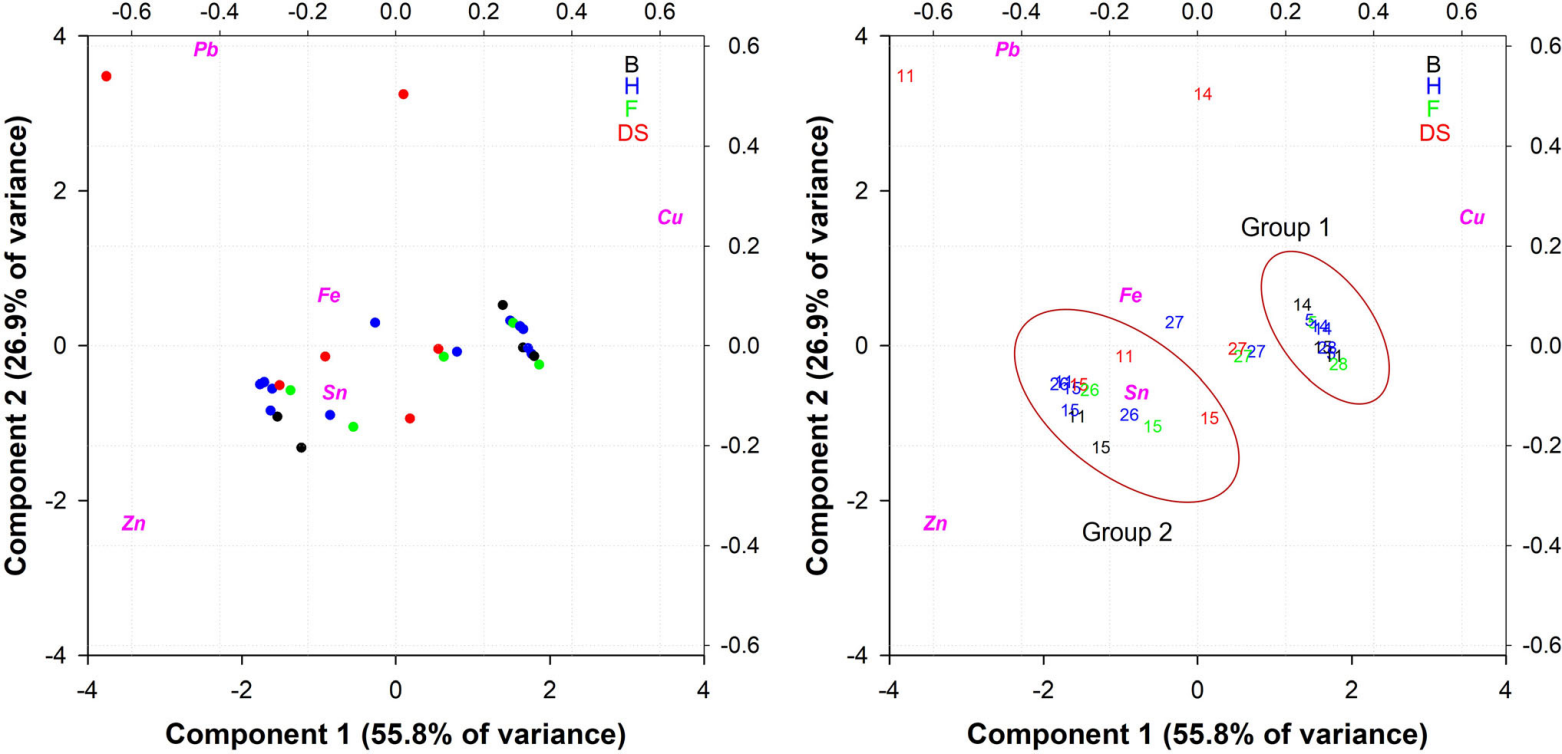
PCA data elaboration on Sesselschreiber statues labelled according to the number of the statue and the statue parts: B = Base; F = Face or cheek; DS = Dagger or sword; H = Hands. Left: Biplot of the ED-XRF raw data. Right: Biplot plot with statue number.

Figure 9 illustrates the PCA analysis (PC1 vs. PC2, 55.8% of total variance) for the statues made by Sesselschreiber. Two distinct clusters can be observed, indicating two separate alloy choices: copper (group 1; statues 5, 14, and 28) and brass (group 2; statues 11, 15, and 26), as previously mentioned. Statue 27 stands out as an exception, being situated between the two main groups and made of a copper-based alloy that contains a smaller quantity of Zn as an alloying element (around 4.8 wt% vs. 16 wt%, which is observed for group 2). The previous principal component analysis (PCA) identified a few outliers appearing in the upper left quadrant: the dagger of statue no. 11 and the scabbard of statue no. 14. Even though these parts noticeably contain higher levels of Pb compared to other parts of the same statues, they also exhibit a high Fe level. Therefore, it is not possible to rule out the possibility of Fe contamination, which may have impacted the interpretation of the point.

The differences in composition of the individual parts of a single statue are not very pronounced, and are within the range of measurement variability. Therefore, it can be concluded that Sesselschreiber used the same alloy for each part of the same statue.

1. However, some notable differences have been observed: the rounded base of the statue no. 11 (brass; group 2) was made of basically pure copper (group 1). In effect, it was one of Sesselschreiber’s statues whose base was cast by Godl (in 1533).
2. The right hand of statue no. 11, the hands of statue no. 15, and the right hand of statue no.27 belong all to group 2 (brass), and exhibit increased levels of Pb in comparison to the standard composition of statues.

## Conclusions

After researching both secondary and primary literature and examining the effigies, it is clear that the available primary sources do not provide a complete understanding of the craft processes involved in creating these impressive examples of Renaissance art in Central Europe. However, it is evident that although the processes at the start of modern craft techniques ultimately led to advancements in the metallurgical sector, a straightforward projection of modern practises onto the past is inadequate in addressing the present matter.

First non-invasive chemical analyses carried out on all the statues provides us with a first insight on the alloys used. The majority of the statues, i.e. 24, was made of rather pure brass. Three statues were made of copper and one of low alloyed copper; they derived from Sesselschreiber’s workshop. It is not possible to differentiate a specific composition or attribute a single alloy to a particular workshop or set of statues. However, the statues by Godl exhibit a significant variation in their Zn content (3.5–22 wt%), which can be attributed to the higher production of statues (17 statues). Sesselschreiber's statues are made of copper/low alloyed copper (Sn, Pb and Zn up to 1 wt. %) or brass with an average zinc content of around 17 wt%. Both Löffler and Vischer use brass with varying zinc amounts (4-22% and 7-22%, respectively). External cast items, such as swords, daggers, hands or faces, often exhibit a slightly different chemical composition than the rest of the statue. The available data does not allow for the differentiation of potential copper sources used by the various artists. However, the absence of Ni in Sesselschreiber's statues suggests the use of a different copper source than the other artists. The four copper statues (nos. 5, 14, 27, and 28) do not show any indication of gilding. However, traces of mercury were discovered on the swords of statues nos. 1 and 4, indicating the use of fire gilding in the local area.

In conclusion, our paper aims to serve as a foundation for future, extensive research on the *Schwarze Mander*. Currently, no technological analyses have been carried out on these Renaissance statues. A thorough study of both the tangible and intangible aspects of craft processes is necessary to fully appreciate these works of art. Consequently, it is indispensable to analyse both the outer and inner surfaces of the statues and experimentally test the hypotheses based on these investigations. This involves researching the utilised moulding clay, joining technology, and the issue of melting and handling large quantities of liquid metal.

It would also be interesting to investigate the relationship between the Tauferer copper mines and the copper used to make the statues. However, this would have to take into account possible cross-contamination from the *Saigerhütten* process, in which copper was desilvered by alloying it with lead. This process was known by 1430 and was well established by the time of Maximilian I. Another very interesting aspect would be to study the patination of the statues, the colour (all black) of which is eponymous for these Renaissance statues. We still do not know when the statues were given this name, nor whether the black colour of the statues is the result of a natural patination process or of artificial patination or the application of specific coatings.

## Supplementary Information

Below is the link to the electronic supplementary material.

Table S1 Results of the XRF-analyses carried out on the statues (sheet 1), including also the standard deviation of the analyses (sheet 2) and the AAS-Analyses published in [12] (sheet 3). The XRF results have been normalized to enable better comparison. (XLSX 70 kb)
